# Genetic Contribution of Emmer Wheat (*Triticum dicoccon* Schrank) to Heat Tolerance of Bread Wheat

**DOI:** 10.3389/fpls.2018.01529

**Published:** 2018-11-20

**Authors:** Smi Ullah, Helen Bramley, Hans Daetwyler, Sang He, Tariq Mahmood, Rebecca Thistlethwaite, Richard Trethowan

**Affiliations:** ^1^School of Life and Environmental Sciences, Plant Breeding Institute, Sydney Institute of Agriculture, The University of Sydney, Narrabri, NSW, Australia; ^2^Agriculture Victoria, AgriBio, Centre for AgriBioscience, Melbourne, VIC, Australia; ^3^School of Applied Systems Biology, La Trobe University, Melbourne, VIC, Australia; ^4^School of Life and Environmental Sciences, Plant Breeding Institute, Sydney Institute of Agriculture, The University of Sydney, Cobbitty, NSW, Australia

**Keywords:** emmer wheat, genetic diversity, genotyping, hexaploid wheat, agronomic traits, heat tolerance

## Abstract

Rising global temperatures cause substantial yield losses in many wheat growing environments. Emmer wheat (*Triticum dicoccon* Schrank), one of the first wheat species domesticated, carries significant variation for tolerance to abiotic stresses. This study identified new genetic variability for high-temperature tolerance in hexaploid progeny derived from crosses with emmer wheat. Eight hexaploid and 11 tetraploid parents were recombined in 43 backcross combinations using the hexaploid as the recurrent parent. A total of 537 emmer-based hexaploid lines were developed by producing approximately 10 doubled haploids on hexaploid like BC_1_F_1_ progeny and subsequent selection for hexaploid morphology. These materials and 17 commercial cultivars and hexaploid recurrent parents were evaluated under two times of sowing in the field, in 2014–2016. The materials were genotyped using a 90K SNP platform and these data were used to estimate the contribution of emmer wheat to the progeny. Significant phenotypic and genetic variation for key agronomical traits including grain yield, TKW and screenings was observed. Many of the emmer derived lines showed improved performance under heat stress (delayed sowing) compared with parents and commercial cultivars. Emmer derived lines were the highest yielding material in both sowing dates. The emmer wheat parent contributed between 1 and 44% of the genome of the derived lines. Emmer derived lines with superior kernel weight and yield generally had a greater genetic contribution from the emmer parent compared to those with lower trait values. The study showed that new genetic variation for key traits such as yield, kernel weight and screenings can be introduced to hexaploid wheat from emmer wheat. These genetic resources should be explored more systematically to stabilize grain yield and quality in a changing climate.

## Introduction

Wheat (*Triticum aestivum*) is a major cereal crop and important for human nutrition worldwide (Fischer et al., 2014; Arzani and Ashraf, 2017). High temperature stress is common in most wheat growing regions of the world, affecting crop productivity, yield stability, and quality (Teixeira et al., 2013; Zhao et al., 2017). Wheat grain characteristics, number, size, and quality, are impacted by heat stress (Lobell et al., 2012; Branlard et al., 2015) and the identification of wheat genotypes with stable yield and quality across a range of environments are important breeding objectives (Dupont et al., 2006; Malik et al., 2013).

Heat tolerance is complex and is controlled by many genes (Richards et al., 2007; Rebetzke et al., 2008; Petrarulo et al., 2009). A number of agronomical, morphological and physiological traits have been associated with heat tolerance of wheat, which include pollen stability and grain set (Lopes and Reynolds, 2012; Mondal et al., 2013; Pinto et al., 2017; Thistlethwaite, 2017). High grain growth rates and larger grain weight have also been linked with improved performance under heat stress (Singha et al., 2006; Dias and Lidon, 2009; Zhang et al., 2014). For breeding programs to be effective, screening should be high-throughput and traits should be easy to measure and highly heritable (Edmeades et al., 2001). Various methods of field-based screening have been evaluated including different sowing dates to study yield and related traits (Mondal et al., 2013; Devasirvatham et al., 2016). Cooper and Podlich (1999) stated that breeding for a target environment is generally effective when selection is made under representative environmental conditions. They concluded that field-based screening should closely represent the most probable conditions experienced by farmers.

Plant breeding has led to genetic erosion due to uniformity requirements and the need to increase productivity (Bharadwaj, 2016). Gradual addition of new genetic variation is needed to sustain the crop improvement process and further enhancements in tolerance to environmental stresses (Dwivedi et al., 2016). The systematic evaluation of genetic resources can define biodiversity patterns, which will facilitate the characterization of allelic variation for yield in stressful environments (Ceccarelli, 2011; Ceccarelli et al., 2013; Bharadwaj, 2016; Dwivedi et al., 2016). However, the addition of diverse allelic variation to applied breeding is problematic (Rasmusson and Phillips, 1997). Thus, to meet short-term breeding objectives; elite germplasm that is pre-adapted to the target environment is used instead of exotic germplasm that requires extensive pre-breeding (Sharma et al., 2013).

For wheat, exotic genetic resources often exhibit better adaptation in stressful climatic conditions (Trethowan and Mujeeb-Kazi, 2008) and contain more diverse genes for stress tolerance (Reynolds et al., 2007; van Ginkel and Ogbonnaya, 2007). These resources include wheat ancestors that can be used to improve the sustainability of wheat production and quality (Nevo, 2014; Arzani and Ashraf, 2017). Emmer wheat (*Triticum dicoccon* Schrank), which is one of the earliest domesticated wheat species and is closely related to modern wheat (Troccoli and Codianni, 2005; Nevo, 2014), has exhibited tolerance to both drought and heat stress (Zaharieva et al., 2010; Nevo, 2014). Moreover, diversity for abiotic and biotic stresses in this tetraploid species can be transferred to commercial wheat cultivars (Xie and Nevo, 2008; Hassan et al., 2016). Introduction of new diversity from all three wheat genomes in emmer-based synthetic wheat produced promising results under stress compared to modern durum wheat synthetic derivatives (Dreisigacker et al., 2008; Trethowan and Mujeeb-Kazi, 2008), especially for drought and high-temperature tolerance (Zaharieva et al., 2010). However, the introduction of emmer-based genetic diversity for heat tolerance in hexaploid wheat has not been fully explored.

This study evaluated a large population of hexaploid wheat genotypes developed through recombination with diverse emmer wheat. These accessions included materials previously identified as tolerant to abiotic stresses (Zaharieva et al., 2010). Genetic diversity for high-temperature tolerance was explored by evaluating the material in the field under managed conditions. It was expected that allelic diversity from emmer wheat would contribute to improved high-temperature stress tolerance in hexaploid wheat.

## Materials and Methods

### Plant Material and Environment

Diverse emmer wheat (*T. dicoccon* Schrank), including accessions identified as stress tolerant by Zaharieva et al. (2010), were crossed and backcrossed with hexaploid bread wheat to transfer A and B genome genetic variation into modern hexaploid wheat. Eight hexaploid and 11 tetraploid parents were recombined in 43 backcross combinations using the hexaploid as the recurrent parent. The pentaploid (AABBD) F_1_ was backcrossed to the hexaploid parent and hexaploid progeny selected based on plant morphology. Doubled haploids were then produced from each hexaploid BC_1_F_1_ plant to produce an average of 10 homozygous lines per plant. A population of approximately 537 doubled haploid genotypes was developed (see Supplementary Table 1 for details) and subsequently evaluated for heat stress, along with their recurrent parents and commercial check cultivars. The parental materials and their pedigrees are reported in Table 1. The genotype Waxwing^∗^2/Kiritati and its progeny were not included in the DNA study as DNA of the parent was not available.

**Table 1:** List of commercial cultivars and parents used to develop emmer-based derivatives evaluated in field studies during 2014–2016.

Checks	Code used in the list of supplementary data	Pedigree	Origin	Ploidy
Suntop	Suntop	Sunco/2^∗^Pastor//SUN-436-E	Australia	6x
Lancer	Lancer	V1184/Chara//Chara/3/Lang	Australia	6x
Spitfire	Spitfire	Drysdale/Kukri	Australia	6x
Mace	Mace	Wyalkatchem/Stylet	Australia	6x
Ega Gregory	Ega Gregory	Pelsart/2^∗^Batavia doubled haploid line	Australia	6x
Sunlin	Sunlin	Sunelg^∗^2//Suneca^∗^3/VPM1	Australia	6x
Orion	Orion	Tatiara/QAL2000	Australia	6x
**Parents**				
PBW 502	PBW 502	W-485/PBW-343//RAJ-1482	INDIA	6x
PBW 550	PBW 550	WH-594/RAJ-3856//W-485	INDIA	6x
DBW-16	DBW-16	RAJ-3765/WR-484//HUW-468	INDIA	6x
DBW-17	DBW-17	CMH-79-A-95/3^∗^CIANO-79//RAJ-3777	INDIA	6x
Sokoll	Sokoll	Pastor/3/Altar-84/AE.SQ(TR.TA)//OPATA-M-85	CIMMYT	6x
Berkut	Berkut	Irena/Baviacora-M-92//Pastor	CIMMYT	6x
Waxwing^∗^2/Kiritati	Waxwing^∗^2/Kiritati	Waxwing^∗^2/Kiritati	CIMMYT	6x
2-49/Cunningham//Kennedy	2-49/Cunningham//Kennedy	2-49/Cunningham//Kennedy	Australia	6x
T.dicocconP194625/ Ae.squarrosa(372)/2/3^∗^Pastor	T.dicocconP194625/ Ae.squarrosa(372)/2/3^∗^Pastor	T.dicocconP194625/ Ae.squarrosa(372)/2/3^∗^Pastor	Australia	6x
*T.dicoccon* 18293 KC75	18293KC75	*Triticum dicoccon* Schrank	AGG	4x
BARI 7531	18341KC75	*Triticum dicoccon* Schrank	AGG	4x
BARI 7533	18343KC75	*Triticum dicoccon* Schrank	AGG	4x
*T.dicoccon* AUS 21758	21758KC75	*Triticum dicoccon* Schrank	AGG	4x
*T dicoccon* AUS 19385	19385KC75	*Triticum dicoccon* Schrank	AGG	4x
*T.dicoccon* C18644	35880MC18644	*Triticum dicoccon* Schrank	CIMMYT	4x
*T dicoccon* 500110	35883M500110	*Triticum dicoccon* Schrank	CIMMYT	4x
*T.dicoccon* C18643	35879MC18643	*Triticum dicoccon* Schrank	CIMMYT	4x
*T.dicoccon* 500132	35888M500132	*Triticum dicoccon* Schrank	CIMMYT	4x
*T.dicoccon* 35884 M500113	35884M500113	*Triticum dicoccon* Schrank	CIMMYT	4x
*T.dicoccon* 500281	35891M500281	*Triticum dicoccon* Schrank	CIMMYT	4x

*Sources: GRIS (Genetic Resources Information System for Wheat and Triticale-CIMMYT): (http://wheatpedigree.net/sort/show/96742). National Variety Trials: (www.nvtonline.com.au)*.

### Experimental Design

Field experiments were sown at the IA Watson Grains Research Centre, The University of Sydney, Narrabri, NSW (30° 20′S 149° 45′E) during the cropping seasons 2014–2016. Experiments were arranged in randomized complete block designs with two replications. Genotypes were sown in plots comprising six rows spaced 33 cm apart and 6 m length, which was reduced to 4 m for harvest. Two experiments were sown adjacent to each other, each year. Experiment 1 (E1, Environment 1) was sown at the optimal sowing date for the region in mid-May. Experiment 2 (E2, Environment 2) was sown 8 weeks after the optimal sowing date.

A set of 200 wheat genotypes from the population were sown in 2014 and a different set of 196 wheat genotypes were sown in 2015. The commercial check cultivars were the same in both years. All genotypes from 2014 to 2015 combined were evaluated in 2016, representing 543 genotypes, including commercial checks, recurrent parents and additional materials. Experiments received supplementary irrigation as required to reduce the confounding effects of drought; particularly in late sown conditions. The experiments were fertilized and managed according to standard industry practices for the region.

### Germplasm Characterization

A common set of traits including days to flowering and physiological maturity, percentage of screenings, thousand kernel weight (TKW) and grain yield was assessed each year following Pask et al. (2012). The grain yield/plot was measured in g/plot and later converted to tons ha^-1^ for all further analyzes. For TKW, five hundred grains were counted using a CONTADOR seed counter (Pfeuffer GmbH, Flugplatzstraβe 70. D-97318 Kitizingen, Germany), then weighed (g) and the result multiplied by 2 to determine TKW. Care was taken to avoid broken grains. Percentage screenings were determined as the amount of split, small sized and shriveled grains in each plot sample (approximately 450–500 g) using an Agtator sieve shaker (Graintec Scientific, QLD, Australia) with 2.0 mm diameter sieves. Screenings were estimated after 40 shakes (standardized for wheat) using the formula; Screenings (%) = (weight of screenings)/(weight of sample) × 100.

### Genetic Analysis

DNA of all genotypes was extracted following the CTAB method proposed by Doyle and Doyle (1990). Five leaves per plot (about 0.5–0.6 g) were collected from random plants of the middle rows of plots during tillering and placed in a 15 mL centrifuge tube containing silica gel to extract the moisture. Tubes containing leaf samples were then kept at room temperature for 7 days to dry the samples for DNA extraction. The material was then genotyped by AgriBio, La Trobe University, VIC, Australia using the Infinium iSelect SNP 90K SNP Assay (Cavanagh et al., 2013; Wang et al., 2014) following the protocol prescribed by the manufacturer. In total 35,267 polymorphic SNP markers were used for further analysis (see Supplementary Data).

### Statistical Analysis

The best linear unbiased estimate (BLUE) of each wheat genotype was calculated using ASReml-R (Gilmour et al., 2009). The phenotypic data was combined across all 3 years within each sowing date and BLUEs calculated for each genotype in each environment. Genotypes and environments were considered fixed terms and ranges/rows within replicates within environments as random terms in the model. GGE bi-plots of the relationships between genotypes and environments were constructed on phenotypic data using the meta-analysis procedure from GenStat version 16 (Payne et al., 2011). GGE bi-plots were produced based on screenings, TKW and grain yield to estimate genotypic stability across both environments. Cluster analysis was performed using the multivariate analysis procedure from GenStat on 90K SNPs data to form similarity matrix and dendrograms were constructed on the basis of genetic distances.

## Results

### Temperature Fluctuations in the Field

Anthesis occurred during the first 2 weeks of September in E1, whereas anthesis was delayed by an average of 4 weeks in E2. The maximum temperature between anthesis and physiological maturity in 2014 was 35°C in E1 and 40.8°C in E2. The respective E1/E2 maximum temperatures in 2015 and 2016 were 35.4/35.6°C and 29.9/36.7°C. High temperature was more pronounced in the later stages of grain filling in all environments (Supplementary Figures 1–3).

### Trait Expression and Stability

**FIGURE 1:**
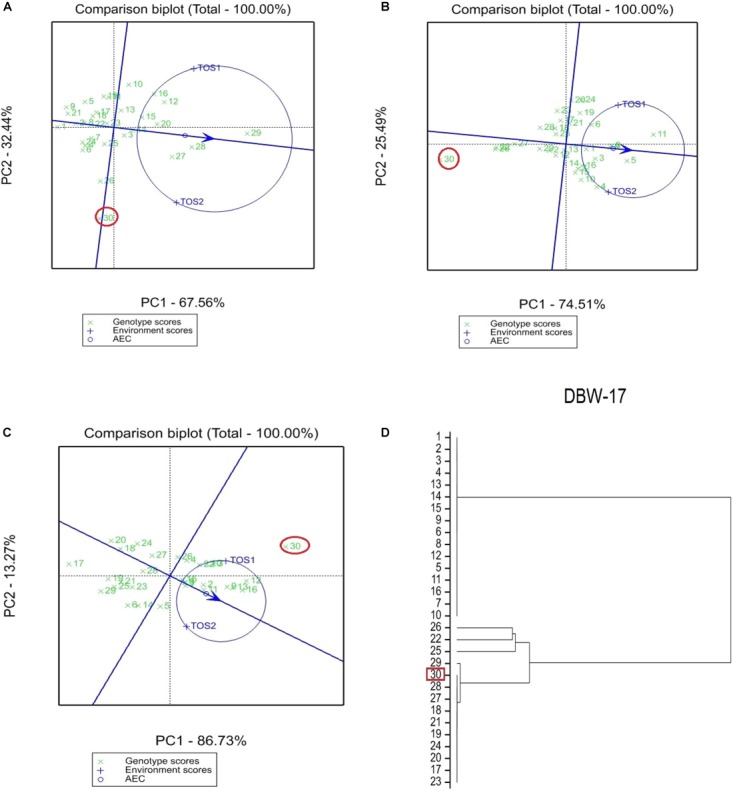
Comparison of recurrent parent DBW-17 (encircled red) and progenies based on mean performance and stability across the two environments (E1, optimal sowing; E2, delayed sowing – heat stressed) for **(A)** percentage screenings, **(B)** thousand kernel weight (TKW), and **(C)** grain yield. A dendrogram constructed using DNA diversity is given in part **(D)**.

**FIGURE 2:**
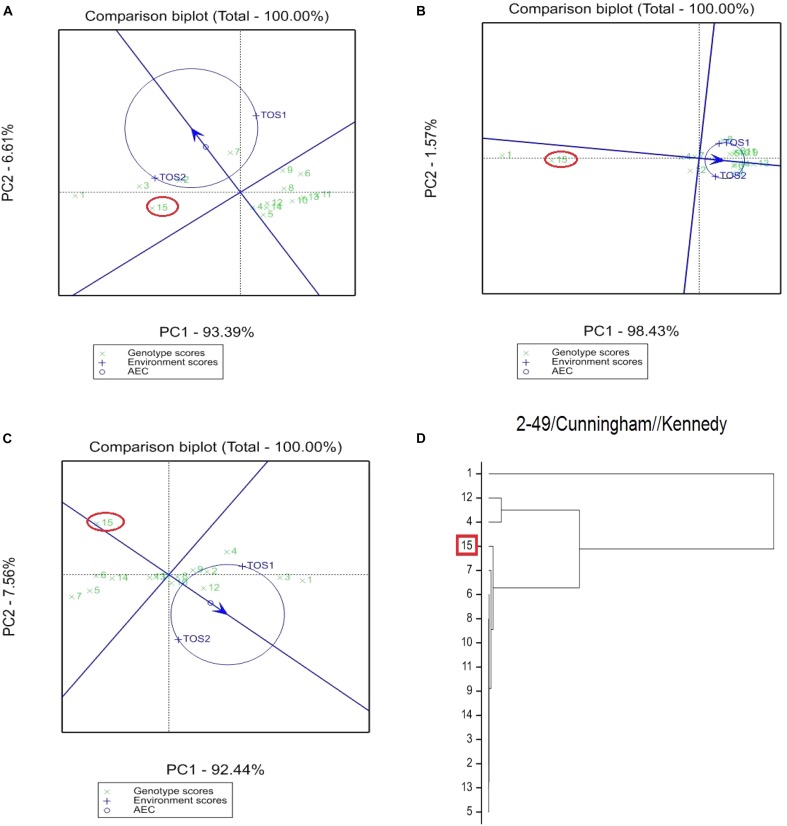
Comparison of recurrent parent 2-49/Cunningham//Kennedy (encircled red) and progenies based on mean performance and stability across the two environments (E1, optimal sowing; E2, delayed sowing – heat stressed) for **(A)** percentage screenings, **(B)** TKW, and **(C)** grain yield. A dendrogram constructed using DNA diversity is given in part **(D)**.

**FIGURE 3:**
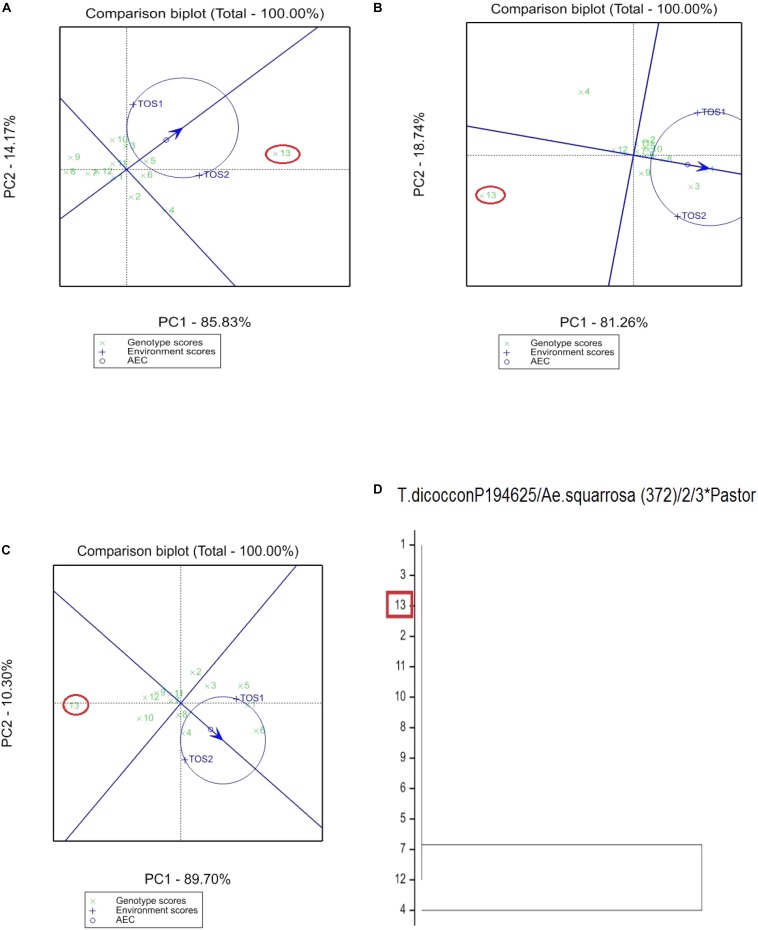
Comparison of recurrent parent T.dicocconP194625/Ae.squarrosa (372)/2/3^∗^Pastor (encircled red) and progenies based on mean performance and stability across the two environments (E1, optimal sowing; E2, delayed sowing – heat stressed) for **(A)** screenings, **(B)** TKW, and **(C)** grain yield. A dendrogram constructed using DNA diversity is given in part **(D)**.

The GGE bi-plot analysis accounted for 100% of the total variation of all traits examined in both environments. There was a significant environmental effect and three typical examples of the responses of the recurrent parent (circled in red) and its derivatives for screenings, TKW and grain yield are presented in Figures 1–3. The responses of all remaining cross combinations are presented in Supplementary Figures 4–8. A dendrogram constructed using DNA diversity (part D in each figure) indicated that many genotypes were closely related to the recurrent parent. This is likely an artifact of the line development process as material was first selected visually for hexaploid type before under-going haploidization and subsequent chromosome doubling. This process skewed the population toward the more stable recurrent parent genotype. The impact of emmer introgression on each trait is presented below.

### Screenings

The derived materials generally had higher screenings than the recurrent parent with the exception of the parents T.dicocconP194625/Ae.squarrosa (372)/2/3^∗^Pastor and 2-49/Cunningham//Kennedy (Figures 2A, 3A). Nevertheless, while the majority of progeny from other crosses had high screenings, there were some with lower screenings than the parents in crosses to PBW 502, DBW-16, and PBW 550 (Table 2). Interestingly, DBW-17 progeny had higher screenings in E1, but reduced screenings when sown late indicating possible tolerance to higher temperatures among some progeny (Figure 1A).

**Table 2:** Emmer derived progeny with superior phenotypic performance compared to their respective recurrent parents and the coefficient of parentage contributed by the emmer parent (COEP) determined by DNA analysis for the two environments (E1, optimal sowing; E2, delayed sowing – heat stressed).

Genotype	COEP	TKW (g)		Screenings (%)		Yield (t/ha)	
		E1	E2	E1	E2	E1	E2
**Berkut**	1.0	47.11	36.13	4.88	15.87	5.24	3.21
Progeny-45	0.44	43.45	28.97	4.82	17.14	5.26	3.15
Progeny-41	0.37	42.28	30.65	4.97	17.12	5.08	3.22
Progeny-26	0.02	42.89	28.98	4.98	16.94	5.30	3.18
Progeny-60	0.02	42.51	30.87	4.17	16.86	5.20	3.17
SE		±0.12	±0.13	±0.02	±0.03	±0.01	±0.002

**Sokoll**	1	38.96	30.88	4.37	16.11	5.05	3.17
Progeny-6	0.31	47.70	37.41	4.81	16.32	5.10	3.14
Progeny-21	0.13	50.57	37.16	4.87	17.08	5.11	3.15
Progeny-57	0.04	45.92	34.28	5.09	16.29	5.17	3.18
Progeny-25	0.03	46.81	36.25	5.20	16.69	5.23	3.21
SE		±0.16	±0.13	±0.02	±0.14	±0.01	±0.02

**PBW 550**	1	43.88	33.52	4.70	16.49	5.18	3.17
Progeny-14	0.35	46.19	33.91	5.29	16.92	5.06	3.18
Progeny-16	0.35	46.12	32.65	5.33	16.62	5.21	3.22
Progeny-13	0.34	47.31	34.22	5.32	16.41	5.07	3.21
Progeny-37	0.03	44.32	34.74	4.99	16.28	5.14	3.24
SE		±0.23	±0.13	±0.05	±0.06	±0.01	±0.002

**PBW 502**	1	48.01	33.84	5.79	16.57	5.04	3.17
Progeny-30	0.34	43.69	33.66	5.08	16.41	5.06	3.19
Progeny-43	0.04	47.86	34.81	5.21	16.43	5.22	3.22
Progeny-19	0.02	48.31	35.21	5.55	16.45	5.02	3.16
Progeny-20	0.01	48.42	34.61	5.63	16.55	5.09	3.19
SE		±0.23	±0.11	±0.05	±0.03	±0.01	±0.002

**DBW-17**	1	40.92	30.55	5.11	17.19	5.17	3.16
Progeny-2	0.27	43.96	32.57	5.50	16.91	5.09	3.18
Progeny-8	0.27	45.65	33.74	5.57	16.97	5.07	3.17
Progeny-11	0.20	46.97	34.31	5.89	16.91	5.09	3.18
Progeny-16	0.16	44.43	33.69	6.24	17.14	5.13	3.20
SE		±0.21	±0.19	±0.06	±0.06	±0.01	±0.004

**DBW-16**	1	45.31	32.72	5.18	17.48	5.03	3.17
Progeny-11	0.38	45.91	34.57	5.40	17.25	5.06	3.13
Progeny-18	0.27	43.76	33.41	5.06	16.79	5.11	3.19
Progeny-17	0.21	43.30	34.39	4.95	16.81	5.13	3.21
Progeny-16	0.17	41.96	29.43	5.54	17.01	5.19	3.18
SE		±0.17	±0.17	±0.06	±0.03	±0.01	±0.002

**2-49/Cunningham// Kennedy**	1	42.51	30.96	4.91	16.93	4.83	3.02
Progeny-1	0.39	40.18	28.60	4.89	17.65	5.11	3.15
Progeny-9	0.06	52.95	38.82	5.50	15.81	4.94	3.11
Progeny-13	0.06	52.76	39.65	5.31	15.57	4.89	3.12
Progeny-11	0.06	52.68	38.48	5.26	15.45	4.91	3.11
SE		0.95	0.78	0.05	0.16	0.02	0.01

**T.dicocconP194625/ Ae.squarrosa**	1	38.69	31.41	5.59	18.28	4.99	3.15
Progeny-4	0.33	43.82	30.25	4.95	17.38	5.11	3.18
Progeny-6	0.02	44.38	33.51	5.27	17.17	5.19	3.19
Progeny-1	0.01	45.83	35.21	5.23	16.92	5.18	3.16
Progeny-8	0.01	44.80	33.99	5.25	16.51	5.11	3.17
SE		±0.46	±0.36	±0.05	±0.12	±0.01	±0.01

*Means underlined are statistically different from the recurrent parent and standard errors (SE) were calculated for each family*.

### Kernel Weight

The derived progeny significantly varied for TKW on the basis of recurrent parent. Crosses to DBW-17 (Figure 1A), 2-49/Cunningham//Kennedy (Figure 2B), T.dicocconP194625/Ae.squarrosa (372)/2/3^∗^Pastor (Figure 3B) and Sokoll produced many progeny with higher TKW than the recurrent parent (Table 2). Some crosses, such as those made to PBW 502, DBW-16, and PBW 550, produced progeny with generally similar TKW, although some with significantly lower TKW were observed. All progeny derived from Berkut had lower TKW than the recurrent parent. The recurrent parent 2-49/Cunningham//Kennedy derived line #9 and #13 had greater TKW and lower screening percentages under normal and stressed environments (Table 2).

### Grain Yield

The grain yield of derived progeny varied significantly with the recurrent parent used. Higher yielding progeny were generally observed in E1 compared with E2. High yielding progeny compared to the recurrent hexaploid parent in E1 were observed for crosses to 2-49/Cunningham//Kennedy (Figure 2C), T.dicoc-conP194625/Ae.squarrosa (372)/2/3^∗^PASTOR (Figure 3C), PBW 502 and Sokoll. Some progeny were high yielding in both environments, including Sokoll and T.dicocconP194625/Ae.squarrosa (372)/2/3^∗^Pastor. Progeny higher yielding than the recurrent parent in E2 only were observed in crosses to DBW-16 and DBW-17 (Figure 1C and Table 2), indicating possible tolerance to higher temperatures. Berkut progeny #26 produced greater yield (5.29 t ha^-1^) under optimal sowing than recurrent hexaploid parent and check cultivars, whereas the PBW 550 derived line #37 produced greater yield (3.24 t ha^-1^) under heat stress.

### Emmer Genetic Contribution and Trait Expression

The range of genetic variation contributed by emmer wheat, determined as the difference between individual progeny and the recurrent parent, varied from 0.01 to 0.44 (Table 2). The greatest emmer wheat contribution was observed for the Berkut progeny #45.

The emmer derived lines in the top and bottom group for each trait in each environment and the emmer parent genetic contribution are given in Table 3. Emmer derived lines in the top 10% based on yield under optimal sowing had an average 11% contribution from the emmer parent compared with only 6% contributed in the bottom 10% (Table 3). Similar trends were observed under heat stress (delayed sowing) where the top 10% of progeny had an emmer contribution of 14% compared with 5% in the bottom group. Grain weight in the top 10% of progeny in both optimal and delayed sowing conditions also had a higher emmer contribution (up to 11%) than the bottom group (up to 6%) (Table 3). However, screenings showed a different emmer contribution depending on sowing date. Under optimal sowing, progeny with lower screenings had a smaller contribution (4%) from the emmer parent compared with 10% in the bottom grouping. This was reversed in late sowing where those with lower screenings had a higher comparative emmer contribution (11 vs. 4%) in the bottom group.

**Table 3:** Means ± SE of the highest and lowest performing progeny and their coefficient of parentage contributed by the emmer parent (COEP) determined by DNA analysis for the two environments (E1, optimal sowing; E2, delayed sowing – heat stressed) for yield, thousand kernel weight (TKW) and percentage screenings compared with the recurrent parents.

Traits	Top 10%	Bottom 10%	Parents	Grand Mean
Yield E1	5.21 ± 0.12	4.95 ± 0.09	5.06 ± 0.02	5.01 ± 0.04
COEP	0.11	0.06	-	-
Yield E2	3.25 ± 0.09	3.09 ± 0.05	3.13 ± 0.02	3.14 ± 0.03
COEP	0.14	0.05	-	-
TKW E1	50.19 ± 0.97	40.66 ± 0.83	43.17 ± 0.87	46.10 ± 0.51
COEP	0.11	0.05	-	-
TKW E2	37.32 ± 0.59	28.80 ± 0.81	32.49 ± 0.63	32.81 ± 0.54
COEP	0.09	0.06	-	-
Screenings E1	4.49 ± 0.31	6.74 ± 0.22	5.05 ± 0.15	6.24 ± 0.19
COEP	0.04	0.10	-	-
Screenings E2	16.09 ± 0.34	17.95 ± 0.32	16.92 ± 0.28	17.11 ± 0.29
COEP	0.11	0.04	-	-

*Standard errors (SE) were calculated using the whole family*.

## Discussion

This study showed that new genetic variation for key traits such as yield, kernel weight, and screenings can be introduced to hexaploid wheat from emmer wheat (Zaharieva et al., 2010; Chandrasekhar et al., 2017). This variation was expressed in both optimally sown and late sown materials, although trait expression under each condition did vary by genotype with some genotypes showing enhanced expression under both conditions. The hexaploid genotypes used as recurrent parents were selected because of their high yield potential and superior performance under late sowing (data not shown); the aim was to find different diversity to that already accumulated in high-yielding and heat tolerant hexaploid wheat genotypes. Interestingly, the theoretical expected 25% contribution of emmer wheat in the single backcross derived progeny was not observed. Instead, this varied between 1 and 44% and was a direct consequence of visual selection for hexaploid appearance and agronomic type, and the subsequent double haploidy process. This also helped to explain the large number of progenies closely related to the recurrent parent obtained from each cross combination. Nevertheless, progeny with <5% deviance from the recurrent parent based on their DNA profiles did produce significantly positive differences in trait values, particularly grain weight and grain yield. It appeared that the larger observed grain weight in the emmer progeny was offset by an increase in smaller grains or screenings as screenings were generally higher in all populations or derived families. It is likely that basal and distal florets failed to fill properly under increased temperature stress in the delayed sowing while those in the center of the spike produced much larger seed.

One of the aims of this research was to identify new sources of allelic variation for high-temperature tolerance as others have reported that useful variation exists in the wild tetraploid gene pool (Wang et al., 2005; Trethowan and Mujeeb-Kazi, 2008; Nevo, 2014). Later sowing increased post-anthesis heat stress, particularly in 2014 and 2015 and this reduced grain yield, lowered TKW and increased screenings. Similar responses to delayed sowing have been observed by others (Tewolde et al., 2006; Mondal et al., 2013). Given the large numbers of lines evaluated, sowing date treatments were not replicated within years; sowing dates were instead replicated across years, thus allowing large numbers of lines to be evaluated with replication in each environment each year. The observed reduction in grain yield under heat stress is most likely associated with reduced starch accumulation, which reduced grain size and weight (Farooq et al., 2011). However, as many of the progeny derived from a range of crosses maintained their grain weight and produced superior yield under heat stress, it can be concluded that useful variation was introduced from emmer wheat.

Thousand kernel weight was positively associated with yield and generally had a higher heritability than yield in earlier studies, making it an ideal target for indirect selection (Cossani and Reynolds, 2015; Pinto et al., 2017). The progeny of two recurrent parents, 2-49/Cunningham//Kennedy and T.dicocconP194625/Ae.squarrosa, had consistently higher kernel weight than their recurrent parents under heat stress. Unlike the progeny of other recurrent parents with equally higher kernel weight, they produced relatively low screenings, indicating an ability to fill all grains within the spike under stress. These materials express genuine high-temperature tolerance. Earlier studies, including that by Chandrasekhar et al. (2017), found that high TKW was linked to high-temperature tolerance; however, as screenings were not presented in that earlier work it is difficult to determine if this tolerance was equivalent.

## Author Contributions

SU designed the experiments, collected the data, analyzed the results, and wrote this article. HB and TM as associated research supervisors facilitated for planning and conducting the research. HD and SH helped in genetic analysis. RJT assisted in phenotyping. RT as a primary research supervisor aided in material development, and strategy advice. All the authors read and approved the final manuscript.

## Conflict of Interest Statement

The authors declare that the research was conducted in the absence of any commercial or financial relationships that could be construed as a potential conflict of interest.
