# Supplementary material for: Genetic Contribution of Emmer Wheat (Triticum dicoccon Schrank) to Heat Tolerance of Bread Wheat
Source: Front Plant Sci. 2018 Nov 20;9:1529. doi: 10.3389/fpls.2018.01529 (PMC6257323; doi:10.3389/fpls.2018.01529)
Supplement: Supplementary file 1 [file Data_Sheet_1.pdf]

Supplementary Material

Genetic Contribution of Emmer Wheat (*Triticum dicoccon* Schrank) to Heat Tolerance of Bread Wheat

Smi Ullah[1*,] Helen Bramley[1], Hans Daetwyler[2,3], Sang He[2], Tariq Mahmood[4], Rebecca Thistlethwaite[1] and Richard Trethowan[1,4]

[1] School of Life and Environmental Sciences, Plant Breeding Institute, Sydney Institute of Agriculture, The University of Sydney, Narrabri, NSW, Australia, [2] Agriculture Victoria, AgriBio, Centre for AgriBioscience, Bundoora 3083, VIC, Australia, [3] School of Applied Systems Biology, La Trobe University, Bundoora 3086, VIC, Australia, [4] School of Life and Environmental Sciences, Plant Breeding Institute, Sydney Institute of Agriculture, The University of Sydney, Cobbitty, NSW, Australia

Correspondence

Corresponding author; Smi Ullah
Email Address; smi.ullah@sydney.edu.au

Supplementary Table 1. List of recurrent hexaploid parents and progenies (a-h) used for experiments

(a) BERKUT and progenies

ID#	Designation	Pedigree
1	PBI09C001-BC-DH35	BERKUT/2/BERKUT / 35880 M C18644
2	PBI09C001-BC-DH43	BERKUT/2/BERKUT / 35880 M C18644
3	PBI09C001-BC-DH63	BERKUT/2/BERKUT / 35880 M C18644
4	PBI09C001-BC-DH70	BERKUT/2/BERKUT / 35880 M C18644
5	PBI09C001-BC-DH96	BERKUT/2/BERKUT / 35880 M C18644
6	PBI09C002-BC-DH3	BERKUT/2/BERKUT / 35891 M500281
7	PBI09C002-BC-DH4	BERKUT/2/BERKUT / 35891 M500281
8	PBI09C002-BC-DH16	BERKUT/2/BERKUT / 35891 M500281
9	PBI09C002-BC-DH17	BERKUT/2/BERKUT / 35891 M500281
10	PBI09C003-BC-DH1	BERKUT/2/BERKUT / 35879 M C18643
11	PBI09C003-BC-DH2	BERKUT/2/BERKUT / 35879 M C18643
12	PBI09C003-BC-DH3	BERKUT/2/BERKUT / 35879 M C18643
13	PBI09C003-BC-DH4	BERKUT/2/BERKUT / 35879 M C18643
14	PBI09C004-BC-DH4	BERKUT/2/BERKUT / 35883 M500110
15	PBI09C004-BC-DH9	BERKUT/2/BERKUT / 35883 M500110
16	PBI09C004-BC-DH10	BERKUT/2/BERKUT / 35883 M500110
17	PBI09C004-BC-DH14	BERKUT/2/BERKUT / 35883 M500110
18	PBI09C004-BC-DH16	BERKUT/2/BERKUT / 35883 M500110
19	PBI09C004-BC-DH17	BERKUT/2/BERKUT / 35883 M500110
20	PBI09C004-BC-DH27	BERKUT/2/BERKUT / 35883 M500110
21	PBI09C004-BC-DH32	BERKUT/2/BERKUT / 35883 M500110
22	PBI09C004-BC-DH33	BERKUT/2/BERKUT / 35883 M500110
23	PBI09C004-BC-DH34	BERKUT/2/BERKUT / 35883 M500110
24	PBI09C004-BC-DH36	BERKUT/2/BERKUT / 35883 M500110
25	PBI09C004-BC-DH39	BERKUT/2/BERKUT / 35883 M500110
26	PBI09C004-BC-DH43	BERKUT/2/BERKUT / 35883 M500110
27	PBI09C004-BC-DH44	BERKUT/2/BERKUT / 35883 M500110

28	PBI09C004-BC-DH55	BERKUT/2/BERKUT / 35883 M500110
29	PBI09C004-BC-DH60	BERKUT/2/BERKUT / 35883 M500110
30	PBI09C004-BC-DH61	BERKUT/2/BERKUT / 35883 M500110
31	PBI09C004-BC-DH62	BERKUT/2/BERKUT / 35883 M500110
32	PBI09C004-BC-DH63	BERKUT/2/BERKUT / 35883 M500110
33	PBI09C004-BC-DH70	BERKUT/2/BERKUT / 35883 M500110
34	PBI09C004-BC-DH73	BERKUT/2/BERKUT / 35883 M500110
35	PBI09C004-BC-DH82	BERKUT/2/BERKUT / 35883 M500110
36	PBI09C004-BC-DH86	BERKUT/2/BERKUT / 35883 M500110
37	PBI09C004-BC-DH87	BERKUT/2/BERKUT / 35883 M500110
38	PBI09C004-BC-DH89	BERKUT/2/BERKUT / 35883 M500110
39	PBI09C004-BC-DH94	BERKUT/2/BERKUT / 35883 M500110
40	PBI09C004-BC-DH100	BERKUT/2/BERKUT / 35883 M500110
41	PBI09C004-BC-DH105	BERKUT/2/BERKUT / 35883 M500110
42	PBI09C004-BC-DH108	BERKUT/2/BERKUT / 35883 M500110
43	PBI09C004-BC-DH109	BERKUT/2/BERKUT / 35883 M500110
44	PBI09C004-BC-DH110	BERKUT/2/BERKUT / 35883 M500110
45	PBI09C004-BC-DH115	BERKUT/2/BERKUT / 35883 M500110
46	PBI09C004-BC-DH116	BERKUT/2/BERKUT / 35883 M500110
47	PBI09C004-BC-DH123	BERKUT/2/BERKUT / 35883 M500110
48	PBI09C004-BC-DH126	BERKUT/2/BERKUT / 35883 M500110
49	PBI09C004-BC-DH127	BERKUT/2/BERKUT / 35883 M500110
50	PBI09C001-BC-DH1	BERKUT/2/BERKUT / 35880 M C18644
51	PBI09C001-BC-DH33	BERKUT/2/BERKUT / 35880 M C18644
52	PBI09C001-BC-DH46	BERKUT/2/BERKUT / 35880 M C18644
53	PBI09C001-BC-DH58	BERKUT/2/BERKUT / 35880 M C18644
54	PBI09C001-BC-DH61	BERKUT/2/BERKUT / 35880 M C18644
55	PBI09C001-BC-DH64	BERKUT/2/BERKUT / 35880 M C18644
56	PBI09C001-BC-DH79	BERKUT/2/BERKUT / 35880 M C18644
57	PBI09C001-BC-DH8	BERKUT/2/BERKUT / 35880 M C18644
58	PBI09C001-BC-DH80	BERKUT/2/BERKUT / 35880 M C18644
59	PBI09C001-BC-DH86	BERKUT/2/BERKUT / 35880 M C18644
60	PBI09C001-BC-DH89	BERKUT/2/BERKUT / 35880 M C18644
61	PBI09C001-BC-DH9	BERKUT/2/BERKUT / 35880 M C18644
62	PBI09C001-BC-DH98	BERKUT/2/BERKUT / 35880 M C18644
63	PBI09C002-BC-DH1	BERKUT/2/BERKUT / 35891 M500281
64	PBI09C002-BC-DH20	BERKUT/2/BERKUT / 35891 M500281
65	PBI09C002-BC-DH5	BERKUT/2/BERKUT / 35891 M500281
66	PBI09C002-BC-DH6	BERKUT/2/BERKUT / 35891 M500281
67	PBI09C002-BC-DH8	BERKUT/2/BERKUT / 35891 M500281
68	PBI09C004-BC-DH1	BERKUT/2/BERKUT / 35883 M500110
69	PBI09C004-BC-DH106	BERKUT/2/BERKUT / 35883 M500110
70	PBI09C004-BC-DH117	BERKUT/2/BERKUT / 35883 M500110
71	PBI09C004-BC-DH118	BERKUT/2/BERKUT / 35883 M500110
72	PBI09C004-BC-DH23	BERKUT/2/BERKUT / 35883 M500110
73	PBI09C004-BC-DH24	BERKUT/2/BERKUT / 35883 M500110
74	PBI09C004-BC-DH51	BERKUT/2/BERKUT / 35883 M500110
75	PBI09C004-BC-DH74	BERKUT/2/BERKUT / 35883 M500110
76	PBI09C004-BC-DH76	BERKUT/2/BERKUT / 35883 M500110
77	PBI09C004-BC-DH78	BERKUT/2/BERKUT / 35883 M500110
78	PBI09C001-BC-DH10	BERKUT/2/BERKUT / 35880 M C18644
79	PBI09C001-BC-DH100	BERKUT/2/BERKUT / 35880 M C18644
80	PBI09C001-BC-DH14	BERKUT/2/BERKUT / 35880 M C18644
81	PBI09C001-BC-DH16	BERKUT/2/BERKUT / 35880 M C18644
82	PBI09C001-BC-DH17	BERKUT/2/BERKUT / 35880 M C18644
83	PBI09C001-BC-DH25	BERKUT/2/BERKUT / 35880 M C18644
84	PBI09C001-BC-DH26	BERKUT/2/BERKUT / 35880 M C18644
85	PBI09C001-BC-DH30	BERKUT/2/BERKUT / 35880 M C18644

86	PBI09C001-BC-DH29	BERKUT/2/BERKUT / 35880 M C18644
87	PBI09C001-BC-DH38	BERKUT/2/BERKUT / 35880 M C18644
88	PBI09C001-BC-DH44	BERKUT/2/BERKUT / 35880 M C18644
89	PBI09C001-BC-DH45	BERKUT/2/BERKUT / 35880 M C18644
90	PBI09C001-BC-DH47	BERKUT/2/BERKUT / 35880 M C18644
91	PBI09C001-BC-DH49	BERKUT/2/BERKUT / 35880 M C18644
92	PBI09C001-BC-DH51	BERKUT/2/BERKUT / 35880 M C18644
93	PBI09C001-BC-DH52	BERKUT/2/BERKUT / 35880 M C18644
94	PBI09C001-BC-DH54	BERKUT/2/BERKUT / 35880 M C18644
95	PBI09C001-BC-DH60	BERKUT/2/BERKUT / 35880 M C18644
96	PBI09C001-BC-DH67	BERKUT/2/BERKUT / 35880 M C18644
97	PBI09C001-BC-DH68	BERKUT/2/BERKUT / 35880 M C18644
98	PBI09C001-BC-DH69	BERKUT/2/BERKUT / 35880 M C18644
99	PBI09C001-BC-DH7	BERKUT/2/BERKUT / 35880 M C18644
100	PBI09C001-BC-DH71	BERKUT/2/BERKUT / 35880 M C18644
101	PBI09C001-BC-DH73	BERKUT/2/BERKUT / 35880 M C18644
102	BERKUT	BERKUT

(b) 2-49/CUNNINGHAM//KENNEDY and progenies

ID#	Designation	Pedigree
1	PBI09C016-BC-DH2	2-49/CUNNINGHAM//KENNEDY/4/2-49/CUNNINGHAM//KENNEDY/3/35880 M C18644
2	PBI09C018-BC-DH21	2-49/CUNNINGHAM//KENNEDY/4/2-49/CUNNINGHAM//KENNEDY/3/35883 M500110
3	PBI09C018-BC-DH25	2-49/CUNNINGHAM//KENNEDY/4/2-49/CUNNINGHAM//KENNEDY/3/35883 M500110
4	PBI09C016-BC-DH4	2-49/CUNNINGHAM//KENNEDY/4/2-49/CUNNINGHAM//KENNEDY /3/ 35880 M C18644
5	PBI09C016-BC-DH7	2-49/CUNNINGHAM//KENNEDY/4/2-49/CUNNINGHAM//KENNEDY /3/ 35880 M C18644
6	PBI09C018-BC-DH11	2-49/CUNNINGHAM//KENNEDY/4/2-49/CUNNINGHAM//KENNEDY /3/ 35883 M500110
7	PBI09C018-BC-DH2	2-49/CUNNINGHAM//KENNEDY/4/2-49/CUNNINGHAM//KENNEDY /3/ 35883 M500110
8	PBI09C018-BC-DH20	2-49/CUNNINGHAM//KENNEDY/4/2-49/CUNNINGHAM//KENNEDY /3/ 35883 M500110
9	PBI09C018-BC-DH29	2-49/CUNNINGHAM//KENNEDY/4/2-49/CUNNINGHAM//KENNEDY /3/ 35883 M500110
10	PBI09C018-BC-DH4	2-49/CUNNINGHAM//KENNEDY/4/2-49/CUNNINGHAM//KENNEDY /3/ 35883 M500110
11	PBI09C018-BC-DH5	2-49/CUNNINGHAM//KENNEDY/4/2-49/CUNNINGHAM//KENNEDY /3/ 35883 M500110
12	PBI09C018-BC-DH6	2-49/CUNNINGHAM//KENNEDY/4/2-49/CUNNINGHAM//KENNEDY /3/ 35883 M500110
13	PBI09C018-BC-DH8	2-49/CUNNINGHAM//KENNEDY/4/2-49/CUNNINGHAM//KENNEDY /3/ 35883 M500110
14	PBI09C018-BC-DH9	2-49/CUNNINGHAM//KENNEDY/4/2-49/CUNNINGHAM//KENNEDY /3/ 35883 M500110
15	2-49/CUNNINGHAM//KENNEDY	2-49/CUNNINGHAM//KENNEDY

(c) SOKOLL and progenies

ID#	Designation	Pedigree
1	PBI09C008-BC-DH2	SOKOLL/2/SOKOLL / 35883 M500110
2	PBI09C008-BC-DH3	SOKOLL/2/SOKOLL / 35883 M500110
3	PBI09C008-BC-DH5	SOKOLL/2/SOKOLL / 35883 M500110
4	PBI09C008-BC-DH6	SOKOLL/2/SOKOLL / 35883 M500110

5	PBI09C008-BC-DH11	SOKOLL/2/SOKOLL / 35883 M500110
6	PBI09C008-BC-DH13	SOKOLL/2/SOKOLL / 35883 M500110
7	PBI09C009-BC-DH9	SOKOLL/2/SOKOLL / 35888 M 500132
8	PBI09C009-BC-DH11	SOKOLL/2/SOKOLL / 35888 M 500132
9	PBI09C009-BC-DH12	SOKOLL/2/SOKOLL / 35888 M 500132
10	PBI09C009-BC-DH13	SOKOLL/2/SOKOLL / 35888 M 500132
11	PBI09C009-BC-DH14	SOKOLL/2/SOKOLL / 35888 M 500132
12	PBI09C009-BC-DH15	SOKOLL/2/SOKOLL / 35888 M 500132
13	PBI09C009-BC-DH19	SOKOLL/2/SOKOLL / 35888 M 500132
14	PBI09C009-BC-DH20	SOKOLL/2/SOKOLL / 35888 M 500132
15	PBI09C009-BC-DH21	SOKOLL/2/SOKOLL / 35888 M 500132
16	PBI09C009-BC-DH23	SOKOLL/2/SOKOLL / 35888 M 500132
17	PBI09C009-BC-DH27	SOKOLL/2/SOKOLL / 35888 M 500132
18	PBI09C009-BC-DH34	SOKOLL/2/SOKOLL / 35888 M 500132
19	PBI09C009-BC-DH35	SOKOLL/2/SOKOLL / 35888 M 500132
20	PBI09C009-BC-DH36	SOKOLL/2/SOKOLL / 35888 M 500132
21	PBI09C009-BC-DH37	SOKOLL/2/SOKOLL / 35888 M 500132
22	PBI09C009-BC-DH44	SOKOLL/2/SOKOLL / 35888 M 500132
23	PBI09C009-BC-DH48	SOKOLL/2/SOKOLL / 35888 M 500132
24	PBI09C009-BC-DH49	SOKOLL/2/SOKOLL / 35888 M 500132
25	PBI09C009-BC-DH51	SOKOLL/2/SOKOLL / 35888 M 500132
26	PBI09C009-BC-DH58	SOKOLL/2/SOKOLL / 35888 M 500132
27	PBI09C009-BC-DH60	SOKOLL/2/SOKOLL / 35888 M 500132
28	PBI09C009-BC-DH62	SOKOLL/2/SOKOLL / 35888 M 500132
29	PBI09C009-BC-DH65	SOKOLL/2/SOKOLL / 35888 M 500132
30	PBI09C009-BC-DH66	SOKOLL/2/SOKOLL / 35888 M 500132
31	PBI09C009-BC-DH67	SOKOLL/2/SOKOLL / 35888 M 500132
32	PBI09C009-BC-DH68	SOKOLL/2/SOKOLL / 35888 M 500132
33	PBI09C009-BC-DH70	SOKOLL/2/SOKOLL / 35888 M 500132
34	PBI09C009-BC-DH72	SOKOLL/2/SOKOLL / 35888 M 500132
35	PBI09C009-BC-DH74	SOKOLL/2/SOKOLL / 35888 M 500132
36	PBI09C009-BC-DH77	SOKOLL/2/SOKOLL / 35888 M 500132
37	PBI09C009-BC-DH78	SOKOLL/2/SOKOLL / 35888 M 500132
38	PBI09C009-BC-DH80	SOKOLL/2/SOKOLL / 35888 M 500132
39	PBI09C009-BC-DH81	SOKOLL/2/SOKOLL / 35888 M 500132
40	PBI09C009-BC-DH82	SOKOLL/2/SOKOLL / 35888 M 500132
41	PBI09C009-BC-DH87	SOKOLL/2/SOKOLL / 35888 M 500132
42	PBI09C009-BC-DH88	SOKOLL/2/SOKOLL / 35888 M 500132
43	PBI09C009-BC-DH93	SOKOLL/2/SOKOLL / 35888 M 500132
44	PBI09C009-BC-DH94	SOKOLL/2/SOKOLL / 35888 M 500132
45	PBI09C009-BC-DH96	SOKOLL/2/SOKOLL / 35888 M 500132
46	PBI09C009-BC-DH99	SOKOLL/2/SOKOLL / 35888 M 500132
47	PBI09C009-BC-DH100	SOKOLL/2/SOKOLL / 35888 M 500132
48	PBI09C010-BC-DH11	SOKOLL/2/SOKOLL / 35879 M C18643
49	PBI09C008-BC-DH1	SOKOLL/2/SOKOLL / 35883 M500110
50	PBI09C008-BC-DH17	SOKOLL/2/SOKOLL / 35883 M500110
51	PBI09C008-BC-DH19	SOKOLL/2/SOKOLL / 35883 M500110
52	PBI09C008-BC-DH20	SOKOLL/2/SOKOLL / 35883 M500110
53	PBI09C008-BC-DH23	SOKOLL/2/SOKOLL / 35883 M500110
54	PBI09C008-BC-DH26	SOKOLL/2/SOKOLL / 35883 M500110
55	PBI09C008-BC-DH30	SOKOLL/2/SOKOLL / 35883 M500110
56	PBI09C008-BC-DH31	SOKOLL/2/SOKOLL / 35883 M500110
57	PBI09C008-BC-DH32	SOKOLL/2/SOKOLL / 35883 M500110
58	PBI09C008-BC-DH35	SOKOLL/2/SOKOLL / 35883 M500110
59	PBI09C008-BC-DH39	SOKOLL/2/SOKOLL / 35883 M500110
60	PBI09C008-BC-DH40	SOKOLL/2/SOKOLL / 35883 M500110
61	PBI09C008-BC-DH7	SOKOLL/2/SOKOLL / 35883 M500110
62	PBI09C008-BC-DH8	SOKOLL/2/SOKOLL / 35883 M500110

63	PBI09C009-BC-DH1	SOKOLL/2/SOKOLL / 35888 M 500132
64	PBI09C009-BC-DH17	SOKOLL/2/SOKOLL / 35888 M 500132
65	PBI09C009-BC-DH2	SOKOLL/2/SOKOLL / 35888 M 500132
66	PBI09C009-BC-DH25	SOKOLL/2/SOKOLL / 35888 M 500132
67	PBI09C009-BC-DH29	SOKOLL/2/SOKOLL / 35888 M 500132
68	PBI09C009-BC-DH30	SOKOLL/2/SOKOLL / 35888 M 500132
69	PBI09C009-BC-DH52	SOKOLL/2/SOKOLL / 35888 M 500132
70	PBI09C009-BC-DH57	SOKOLL/2/SOKOLL / 35888 M 500132
71	PBI09C009-BC-DH71	SOKOLL/2/SOKOLL / 35888 M 500132
72	PBI09C009-BC-DH76	SOKOLL/2/SOKOLL / 35888 M 500132
73	PBI09C009-BC-DH84	SOKOLL/2/SOKOLL / 35888 M 500132
74	PBI09C009-BC-DH86	SOKOLL/2/SOKOLL / 35888 M 500132
75	PBI09C009-BC-DH89	SOKOLL/2/SOKOLL / 35888 M 500132
76	PBI09C010-BC-DH1	SOKOLL/2/SOKOLL / 35879 M C18643
77	PBI09C010-BC-DH10	SOKOLL/2/SOKOLL / 35879 M C18643
78	PBI09C010-BC-DH13	SOKOLL/2/SOKOLL / 35879 M C18643
79	PBI09C010-BC-DH15	SOKOLL/2/SOKOLL / 35879 M C18643
80	PBI09C010-BC-DH18	SOKOLL/2/SOKOLL / 35879 M C18643
81	PBI09C010-BC-DH19	SOKOLL/2/SOKOLL / 35879 M C18643
82	PBI09C010-BC-DH3	SOKOLL/2/SOKOLL / 35879 M C18643
83	PBI09C010-BC-DH4	SOKOLL/2/SOKOLL / 35879 M C18643
84	PBI09C010-BC-DH7	SOKOLL/2/SOKOLL / 35879 M C18643
85	PBI09C010-BC-DH8	SOKOLL/2/SOKOLL / 35879 M C18643
86	PBI09C010-BC-DH9	SOKOLL/2/SOKOLL / 35879 M C18643
87	SOKOLL	SOKOLL

(d) T.DICOCCONP194625/AE.SQUARROSA(372)/2/3*PASTOR and progenies

ID#	Designation	Pedigree
1	PBI09C021-BC-DH1	T.DICOCCONP194625/AE.SQUARROSA(372)/2/3*PASTOR /4/T.DICOCCONP194625/AE.SQUARROSA(372)/2/3*PASTOR /3/ 35883 M500110
2	PBI09C021-BC-DH2	T.DICOCCONP194625/AE.SQUARROSA(372)/2/3*PASTOR /4/T.DICOCCONP194625/AE.SQUARROSA(372)/2/3*PASTOR /3/ 35883 M500110
3	PBI09C021-BC-DH8	T.DICOCCONP194625/AE.SQUARROSA(372)/2/3*PASTOR /4/T.DICOCCONP194625/AE.SQUARROSA(372)/2/3*PASTOR /3/ 35883 M500110
4	PBI09C021-BC-DH9	T.DICOCCONP194625/AE.SQUARROSA(372)/2/3*PASTOR /4/T.DICOCCONP194625/AE.SQUARROSA(372)/2/3*PASTOR /3/ 35883 M500110
5	PBI09C021-BC-DH15	T.DICOCCONP194625/AE.SQUARROSA(372)/2/3*PASTOR /4/T.DICOCCONP194625/AE.SQUARROSA(372)/2/3*PASTOR /3/ 35883 M500110
6	PBI09C021-BC-DH16	T.DICOCCONP194625/AE.SQUARROSA(372)/2/3*PASTOR /4/T.DICOCCONP194625/AE.SQUARROSA(372)/2/3*PASTOR /3/ 35883 M500110
7	PBI09C021-BC-DH19	T.DICOCCONP194625/AE.SQUARROSA(372)/2/3*PASTOR /4/T.DICOCCONP194625/AE.SQUARROSA(372)/2/3*PASTOR /3/ 35883 M500110
8	PBI09C021-BC-DH4	T.DICOCCONP194625/AE.SQUARROSA(372)/2/3*PASTOR /4/T.DICOCCONP194625/AE.SQUARROSA(372)/2/3*PASTOR /3/ 35883 M500110
9	PBI09C021-BC-DH5	T.DICOCCONP194625/AE.SQUARROSA(372)/2/3*PASTOR /4/T.DICOCCONP194625/AE.SQUARROSA(372)/2/3*PASTOR /3/ 35883 M500110
10	PBI09C021-BC-DH7	T.DICOCCONP194625/AE.SQUARROSA(372)/2/3*PASTOR /4/T.DICOCCONP194625/AE.SQUARROSA(372)/2/3*PASTOR /3/ 35883 M500110

11	PBI09C023-BC-DH2	T.DICOCCONP194625/AE.SQUARROSA(372)/2/3*PASTOR /4/T.DICOCCONP194625/AE.SQUARROSA(372)/2/3*PASTOR /3/ 35884 M500113
12	PBI09C023-BC-DH3	T.DICOCCONP194625/AE.SQUARROSA(372)/2/3*PASTOR /4/T.DICOCCONP194625/AE.SQUARROSA(372)/2/3*PASTOR /3/ 35884 M500113
13	T.DICOCCONP194625/AE.SQUARROSA	T.DICOCCONP194625/AE.SQUARROSA

(e) PBW 550 and progenies

ID#	Designation	Pedigree
1	PBI09C038-BC-DH3	PBW550 /2/PBW550 / 18293 KC75
2	PBI09C038-BC-DH5	PBW550 /2/PBW550 / 18293 KC75
3	PBI09C038-BC-DH6	PBW550 /2/PBW550 / 18293 KC75
4	PBI09C038-BC-DH11	PBW550 /2/PBW550 / 18293 KC75
5	PBI09C038-BC-DH12	PBW550 /2/PBW550 / 18293 KC75
6	PBI09C038-BC-DH13	PBW550 /2/PBW550 / 18293 KC75
7	PBI09C038-BC-DH14	PBW550 /2/PBW550 / 18293 KC75
8	PBI09C038-BC-DH15	PBW550 /2/PBW550 / 18293 KC75
9	PBI09C038-BC-DH18	PBW550 /2/PBW550 / 18293 KC75
10	PBI09C038-BC-DH20	PBW550 /2/PBW550 / 18293 KC75
11	PBI09C039-BC-DH2	PBW550 /2/PBW550 / 18343 KC75
12	PBI09C039-BC-DH12	PBW550 /2/PBW550 / 18343 KC75
13	PBI09C039-BC-DH13	PBW550 /2/PBW550 / 18343 KC75
14	PBI09C039-BC-DH24	PBW550 /2/PBW550 / 18343 KC75
15	PBI09C039-BC-DH25	PBW550 /2/PBW550 / 18343 KC75
16	PBI09C039-BC-DH37	PBW550 /2/PBW550 / 18343 KC75
17	PBI09C039-BC-DH42	PBW550 /2/PBW550 / 18343 KC75
18	PBI09C039-BC-DH43	PBW550 /2/PBW550 / 18343 KC75
19	PBI09C039-BC-DH45	PBW550 /2/PBW550 / 18343 KC75
20	PBI09C039-BC-DH52	PBW550 /2/PBW550 / 18343 KC75
21	PBI09C039-BC-DH54	PBW550 /2/PBW550 / 18343 KC75
22	PBI09C039-BC-DH57	PBW550 /2/PBW550 / 18343 KC75
23	PBI09C039-BC-DH61	PBW550 /2/PBW550 / 18343 KC75
24	PBI09C039-BC-DH66	PBW550 /2/PBW550 / 18343 KC75
25	PBI09C039-BC-DH68	PBW550 /2/PBW550 / 18343 KC75
26	PBI09C039-BC-DH70	PBW550 /2/PBW550 / 18343 KC75
27	PBI09C039-BC-DH76	PBW550 /2/PBW550 / 18343 KC75
28	PBI09C039-BC-DH77	PBW550 /2/PBW550 / 18343 KC75
29	PBI09C039-BC-DH79	PBW550 /2/PBW550 / 18343 KC75
30	PBI09C039-BC-DH83	PBW550 /2/PBW550 / 18343 KC75
31	PBI09C039-BC-DH86	PBW550 /2/PBW550 / 18343 KC75
32	PBI09C039-BC-DH87	PBW550 /2/PBW550 / 18343 KC75
33	PBI09C039-BC-DH89	PBW550 /2/PBW550 / 18343 KC75
34	PBI09C038-BC-DH23	PBW550 /2/PBW550 / 18293 KC75
35	PBI09C038-BC-DH24	PBW550 /2/PBW550 / 18293 KC75
36	PBI09C038-BC-DH4	PBW550 /2/PBW550 / 18293 KC75
37	PBI09C038-BC-DH7	PBW550 /2/PBW550 / 18293 KC75
38	PBI09C038-BC-DH9	PBW550 /2/PBW550 / 18293 KC75
39	PBI09C039-BC-DH26	PBW550 /2/PBW550 / 18343 KC75
40	PBI09C039-BC-DH40	PBW550 /2/PBW550 / 18343 KC75
41	PBI09C039-BC-DH46	PBW550 /2/PBW550 / 18343 KC75
42	PBI09C039-BC-DH47	PBW550 /2/PBW550 / 18343 KC75
43	PBI09C039-BC-DH53	PBW550 /2/PBW550 / 18343 KC75
44	PBI09C039-BC-DH60	PBW550 /2/PBW550 / 18343 KC75
45	PBI09C039-BC-DH69	PBW550 /2/PBW550 / 18343 KC75
46	PBI09C039-BC-DH73	PBW550 /2/PBW550 / 18343 KC75

47	PBI09C039-BC-DH88	PBW550 /2/PBW550 / 18343 KC75
48	PBI09C039-BC-DH9	PBW550 /2/PBW550 / 18343 KC75
49	PBW550	PBW550

(f) DBW-17 and progenies

ID#	Designation	Pedigree
1	PBI09C048-BC-DH1	DBW17 /2/DBW17 / 21758 KC75
2	PBI09C048-BC-DH3	DBW17 /2/DBW17 / 21758 KC75
3	PBI09C048-BC-DH4	DBW17 /2/DBW17 / 21758 KC75
4	PBI09C048-BC-DH5	DBW17 /2/DBW17 / 21758 KC75
5	PBI09C048-BC-DH7	DBW17 /2/DBW17 / 21758 KC75
6	PBI09C048-BC-DH9	DBW17 /2/DBW17 / 21758 KC75
7	PBI09C048-BC-DH10	DBW17 /2/DBW17 / 21758 KC75
8	PBI09C048-BC-DH11	DBW17 /2/DBW17 / 21758 KC75
9	PBI09C048-BC-DH13	DBW17 /2/DBW17 / 21758 KC75
10	PBI09C048-BC-DH14	DBW17 /2/DBW17 / 21758 KC75
11	PBI09C048-BC-DH15	DBW17 /2/DBW17 / 21758 KC75
12	PBI09C048-BC-DH16	DBW17 /2/DBW17 / 21758 KC75
13	PBI09C048-BC-DH17	DBW17 /2/DBW17 / 21758 KC75
14	PBI09C048-BC-DH18	DBW17 /2/DBW17 / 21758 KC75
15	PBI09C048-BC-DH19	DBW17 /2/DBW17 / 21758 KC75
16	PBI09C048-BC-DH20	DBW17 /2/DBW17 / 21758 KC75
17	PBI09C048-BC-DH25	DBW17 /2/DBW17 / 21758 KC75
18	PBI09C048-BC-DH28	DBW17 /2/DBW17 / 21758 KC75
19	PBI09C048-BC-DH31	DBW17 /2/DBW17 / 21758 KC75
20	PBI09C048-BC-DH35	DBW17 /2/DBW17 / 21758 KC75
21	PBI09C048-BC-DH36	DBW17 /2/DBW17 / 21758 KC75
22	PBI09C048-BC-DH37	DBW17 /2/DBW17 / 21758 KC75
23	PBI09C048-BC-DH38	DBW17 /2/DBW17 / 21758 KC75
24	PBI09C051-BC-DH3	DBW17 /2/DBW17 / 18341 KC75
25	PBI09C049-BC-DH1	DBW17 /2/DBW17 / 19385 KC75
26	PBI09C049-BC-DH4	DBW17 /2/DBW17 / 19385 KC75
27	PBI09C049-BC-DH5	DBW17 /2/DBW17 / 19385 KC75
28	PBI09C049-BC-DH6	DBW17 /2/DBW17 / 19385 KC75
29	PBI09C051-BC-DH4	DBW17 /2/DBW17 / 18341 KC75
30	DBW17	DBW17

(g) DBW-16 and progenies

ID#	Designation	Pedigree
1	PBI09C043-BC-DH4	DBW16 /2/DBW16 / 21758 KC75
2	PBI09C043-BC-DH21	DBW16 /2/DBW16 / 21758 KC75
3	PBI09C043-BC-DH24	DBW16 /2/DBW16 / 21758 KC75
4	PBI09C043-BC-DH26	DBW16 /2/DBW16 / 21758 KC75
5	PBI09C043-BC-DH32	DBW16 /2/DBW16 / 21758 KC75
6	PBI09C043-BC-DH35	DBW16 /2/DBW16 / 21758 KC75
7	PBI09C043-BC-DH39	DBW16 /2/DBW16 / 21758 KC75
8	PBI09C043-BC-DH42	DBW16 /2/DBW16 / 21758 KC75
9	PBI09C043-BC-DH43	DBW16 /2/DBW16 / 21758 KC75
10	PBI09C043-BC-DH45	DBW16 /2/DBW16 / 21758 KC75
11	PBI09C043-BC-DH46	DBW16 /2/DBW16 / 21758 KC75
12	PBI09C043-BC-DH52	DBW16 /2/DBW16 / 21758 KC75
13	PBI09C043-BC-DH56	DBW16 /2/DBW16 / 21758 KC75
14	PBI09C043-BC-DH58	DBW16 /2/DBW16 / 21758 KC75
15	PBI09C043-BC-DH59	DBW16 /2/DBW16 / 21758 KC75
16	PBI09C043-BC-DH60	DBW16 /2/DBW16 / 21758 KC75
17	PBI09C043-BC-DH61	DBW16 /2/DBW16 / 21758 KC75
18	PBI09C043-BC-DH62	DBW16 /2/DBW16 / 21758 KC75

19	PBI09C043-BC-DH63	DBW16 /2/DBW16 / 21758 KC75
20	PBI09C043-BC-DH64	DBW16 /2/DBW16 / 21758 KC75
21	PBI09C043-BC-DH66	DBW16 /2/DBW16 / 21758 KC75
22	PBI09C045-BC-DH1	DBW16 /2/DBW16 / 18341 KC75
23	PBI09C045-BC-DH5	DBW16 /2/DBW16 / 18341 KC75
24	PBI09C045-BC-DH7	DBW16 /2/DBW16 / 18341 KC75
25	PBI09C045-BC-DH11	DBW16 /2/DBW16 / 18341 KC75
26	PBI09C045-BC-DH12	DBW16 /2/DBW16 / 18341 KC75
27	PBI09C045-BC-DH16	DBW16 /2/DBW16 / 18341 KC75
28	PBI09C045-BC-DH19	DBW16 /2/DBW16 / 18341 KC75
29	PBI09C045-BC-DH22	DBW16 /2/DBW16 / 18341 KC75
30	PBI09C045-BC-DH23	DBW16 /2/DBW16 / 18341 KC75
31	PBI09C045-BC-DH24	DBW16 /2/DBW16 / 18341 KC75
32	PBI09C043-BC-DH10	DBW16 /2/DBW16 / 21758 KC75
33	PBI09C043-BC-DH11	DBW16 /2/DBW16 / 21758 KC75
34	PBI09C043-BC-DH12	DBW16 /2/DBW16 / 21758 KC75
35	PBI09C043-BC-DH14	DBW16 /2/DBW16 / 21758 KC75
36	PBI09C043-BC-DH15	DBW16 /2/DBW16 / 21758 KC75
37	PBI09C043-BC-DH22	DBW16 /2/DBW16 / 21758 KC75
38	PBI09C043-BC-DH28	DBW16 /2/DBW16 / 21758 KC75
39	PBI09C043-BC-DH3	DBW16 /2/DBW16 / 21758 KC75
40	PBI09C043-BC-DH31	DBW16 /2/DBW16 / 21758 KC75
41	PBI09C043-BC-DH44	DBW16 /2/DBW16 / 21758 KC75
42	PBI09C043-BC-DH48	DBW16 /2/DBW16 / 21758 KC75
43	PBI09C043-BC-DH51	DBW16 /2/DBW16 / 21758 KC75
44	PBI09C043-BC-DH55	DBW16 /2/DBW16 / 21758 KC75
45	PBI09C045-BC-DH13	DBW16 /2/DBW16 / 18341 KC75
46	PBI09C045-BC-DH15	DBW16 /2/DBW16 / 18341 KC75
47	PBI09C045-BC-DH17	DBW16 /2/DBW16 / 18341 KC75
48	PBI09C045-BC-DH2	DBW16 /2/DBW16 / 18341 KC75
49	PBI09C045-BC-DH20	DBW16 /2/DBW16 / 18341 KC75
50	PBI09C045-BC-DH21	DBW16 /2/DBW16 / 18341 KC75
51	PBI09C045-BC-DH27	DBW16 /2/DBW16 / 18341 KC75
52	PBI09C045-BC-DH28	DBW16 /2/DBW16 / 18341 KC75
53	PBI09C045-BC-DH30	DBW16 /2/DBW16 / 18341 KC75
54	PBI09C045-BC-DH31	DBW16 /2/DBW16 / 18341 KC75
55	PBI09C045-BC-DH4	DBW16 /2/DBW16 / 18341 KC75
56	PBI09C045-BC-DH6	DBW16 /2/DBW16 / 18341 KC75
57	PBI09C043-BC-DH17	DBW16 /2/DBW16 / 21758 KC75
58	PBI09C045-BC-DH9	DBW16 /2/DBW16 / 18341 KC75
59	DBW16	DBW16

(h) PBW 502 and progenies

ID#	Designation	Pedigree
1	PBI09C034-BC-DH1	PBW502 /2/PBW502 / 21758 KC75
2	PBI09C034-BC-DH2	PBW502 /2/PBW502 / 21758 KC75
3	PBI09C034-BC-DH3	PBW502 /2/PBW502 / 21758 KC75
4	PBI09C034-BC-DH4	PBW502 /2/PBW502 / 21758 KC75
5	PBI09C034-BC-DH5	PBW502 /2/PBW502 / 21758 KC75
6	PBI09C034-BC-DH6	PBW502 /2/PBW502 / 21758 KC75
7	PBI09C034-BC-DH7	PBW502 /2/PBW502 / 21758 KC75
8	PBI09C034-BC-DH8	PBW502 /2/PBW502 / 21758 KC75
9	PBI09C034-BC-DH11	PBW502 /2/PBW502 / 21758 KC75
10	PBI09C034-BC-DH15	PBW502 /2/PBW502 / 21758 KC75
11	PBI09C034-BC-DH20	PBW502 /2/PBW502 / 21758 KC75
12	PBI09C034-BC-DH22	PBW502 /2/PBW502 / 21758 KC75
13	PBI09C034-BC-DH26	PBW502 /2/PBW502 / 21758 KC75

14	PBI09C034-BC-DH31	PBW502 /2/PBW502 / 21758 KC75
15	PBI09C034-BC-DH32	PBW502 /2/PBW502 / 21758 KC75
16	PBI09C034-BC-DH36	PBW502 /2/PBW502 / 21758 KC75
17	PBI09C034-BC-DH37	PBW502 /2/PBW502 / 21758 KC75
18	PBI09C034-BC-DH38	PBW502 /2/PBW502 / 21758 KC75
19	PBI09C034-BC-DH40	PBW502 /2/PBW502 / 21758 KC75
20	PBI09C034-BC-DH41	PBW502 /2/PBW502 / 21758 KC75
21	PBI09C034-BC-DH42	PBW502 /2/PBW502 / 21758 KC75
22	PBI09C034-BC-DH43	PBW502 /2/PBW502 / 21758 KC75
23	PBI09C035-BC-DH1	PBW502 /2/PBW502 / 19385 KC75
24	PBI09C035-BC-DH4	PBW502 /2/PBW502 / 19385 KC75
25	PBI09C035-BC-DH6	PBW502 /2/PBW502 / 19385 KC75
26	PBI09C035-BC-DH8	PBW502 /2/PBW502 / 19385 KC75
27	PBI09C035-BC-DH9	PBW502 /2/PBW502 / 19385 KC75
28	PBI09C035-BC-DH10	PBW502 /2/PBW502 / 19385 KC75
29	PBI09C035-BC-DH12	PBW502 /2/PBW502 / 19385 KC75
30	PBI09C035-BC-DH14	PBW502 /2/PBW502 / 19385 KC75
31	PBI09C035-BC-DH15	PBW502 /2/PBW502 / 19385 KC75
32	PBI09C035-BC-DH16	PBW502 /2/PBW502 / 19385 KC75
33	PBI09C035-BC-DH17	PBW502 /2/PBW502 / 19385 KC75
34	PBI09C035-BC-DH18	PBW502 /2/PBW502 / 19385 KC75
35	PBI09C035-BC-DH19	PBW502 /2/PBW502 / 19385 KC75
36	PBI09C035-BC-DH23	PBW502 /2/PBW502 / 19385 KC75
37	PBI09C035-BC-DH29	PBW502 /2/PBW502 / 19385 KC75
38	PBI09C035-BC-DH30	PBW502 /2/PBW502 / 19385 KC75
39	PBI09C035-BC-DH31	PBW502 /2/PBW502 / 19385 KC75
40	PBI09C035-BC-DH33	PBW502 /2/PBW502 / 19385 KC75
41	PBI09C035-BC-DH35	PBW502 /2/PBW502 / 19385 KC75
42	PBI09C035-BC-DH38	PBW502 /2/PBW502 / 19385 KC75
43	PBI09C035-BC-DH39	PBW502 /2/PBW502 / 19385 KC75
44	PBI09C035-BC-DH40	PBW502 /2/PBW502 / 19385 KC75
45	PBI09C035-BC-DH42	PBW502 /2/PBW502 / 19385 KC75
46	PBI09C035-BC-DH43	PBW502 /2/PBW502 / 19385 KC75
47	PBI09C035-BC-DH45	PBW502 /2/PBW502 / 19385 KC75
48	PBI09C035-BC-DH46	PBW502 /2/PBW502 / 19385 KC75
49	PBI09C034-BC-DH17	PBW502 /2/PBW502 / 21758 KC75
50	PBI09C034-BC-DH19	PBW502 /2/PBW502 / 21758 KC75
51	PBI09C034-BC-DH21	PBW502 /2/PBW502 / 21758 KC75
52	PBI09C034-BC-DH23	PBW502 /2/PBW502 / 21758 KC75
53	PBI09C034-BC-DH27	PBW502 /2/PBW502 / 21758 KC75
54	PBI09C034-BC-DH29	PBW502 /2/PBW502 / 21758 KC75
55	PBI09C034-BC-DH30	PBW502 /2/PBW502 / 21758 KC75
56	PBI09C034-BC-DH33	PBW502 /2/PBW502 / 21758 KC75
57	PBI09C034-BC-DH34	PBW502 /2/PBW502 / 21758 KC75
58	PBI09C034-BC-DH9	PBW502 /2/PBW502 / 21758 KC75
59	PBI09C035-BC-DH11	PBW502 /2/PBW502 / 19385 KC75
60	PBI09C035-BC-DH13	PBW502 /2/PBW502 / 19385 KC75
61	PBI09C035-BC-DH2	PBW502 /2/PBW502 / 19385 KC75
62	PBI09C035-BC-DH20	PBW502 /2/PBW502 / 19385 KC75
63	PBI09C035-BC-DH21	PBW502 /2/PBW502 / 19385 KC75
64	PBI09C035-BC-DH22	PBW502 /2/PBW502 / 19385 KC75
65	PBI09C035-BC-DH25	PBW502 /2/PBW502 / 19385 KC75
66	PBI09C035-BC-DH26	PBW502 /2/PBW502 / 19385 KC75
67	PBI09C035-BC-DH28	PBW502 /2/PBW502 / 19385 KC75
68	PBI09C035-BC-DH3	PBW502 /2/PBW502 / 19385 KC75
69	PBI09C035-BC-DH37	PBW502 /2/PBW502 / 19385 KC75
70	PBI09C035-BC-DH41	PBW502 /2/PBW502 / 19385 KC75
71	PBI09C035-BC-DH7	PBW502 /2/PBW502 / 19385 KC75

72	PBW502	PBW502

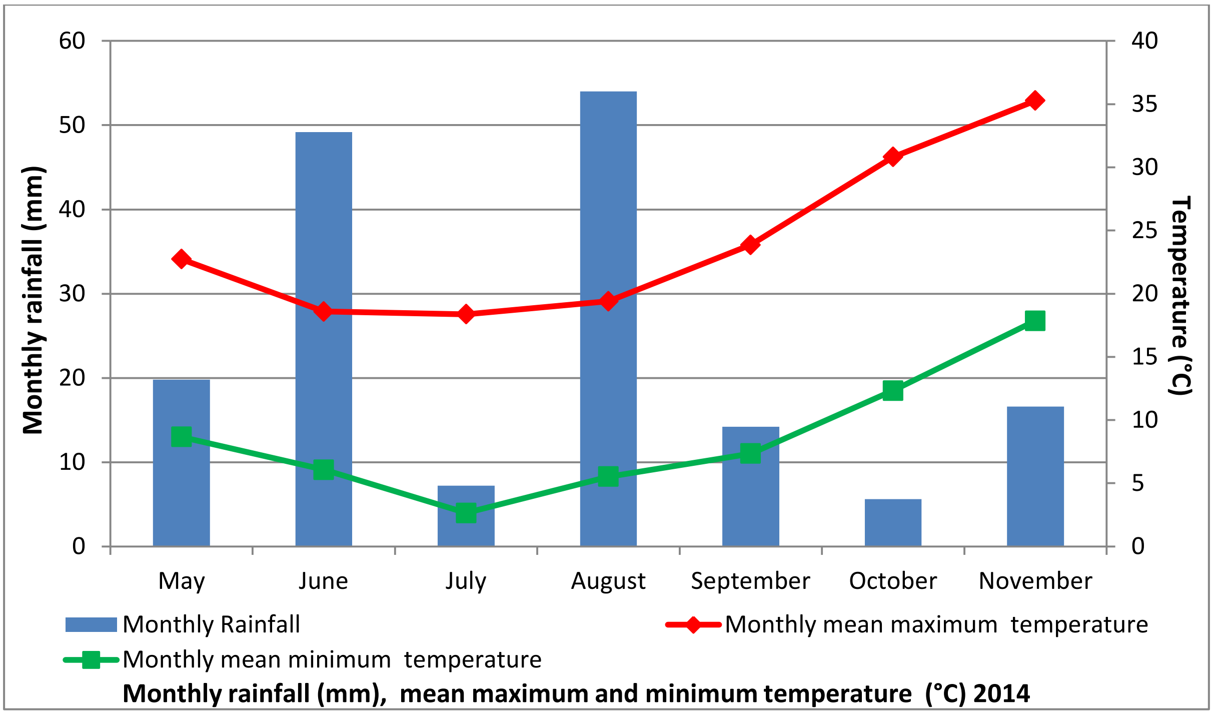

Supplementary Figure 1. Monthly rainfall (mm), and mean maximum and minimum temperatures at Narrabri during 2014 growing season.

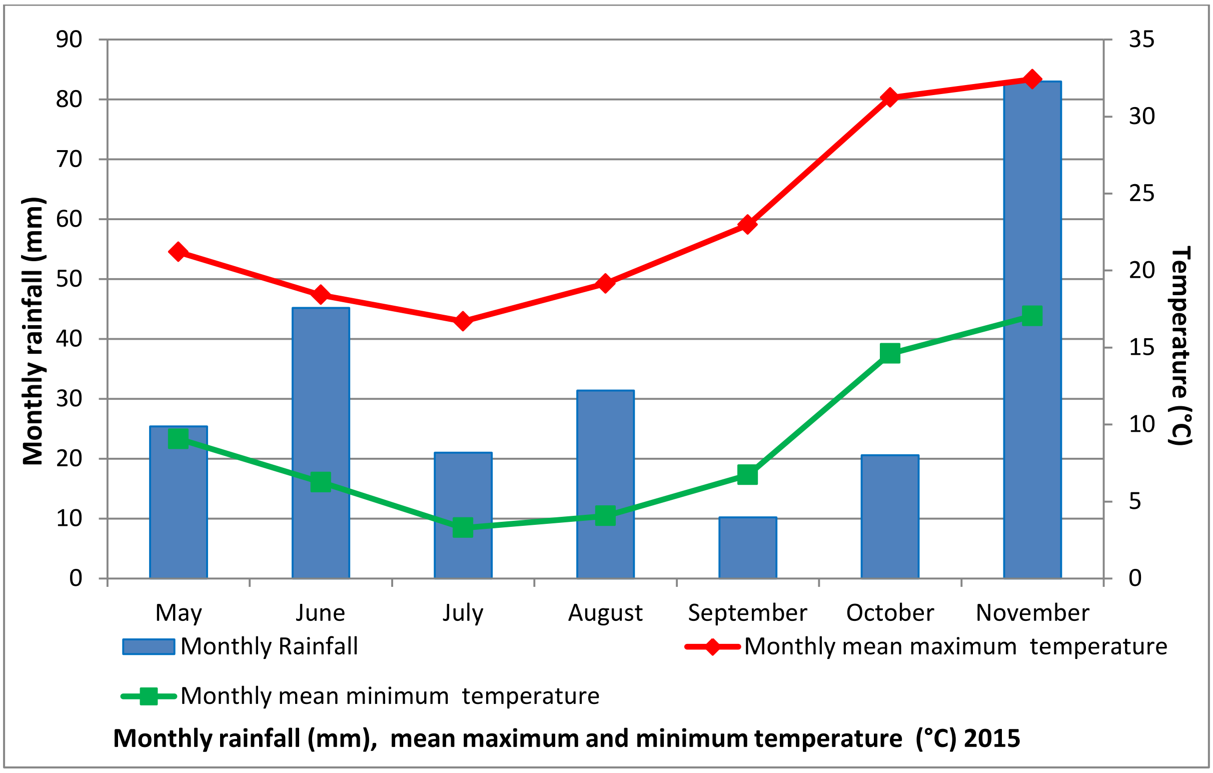

Supplementary Figure 2. Monthly rainfall (mm), and mean maximum and minimum temperatures at Narrabri during 2015 growing season.

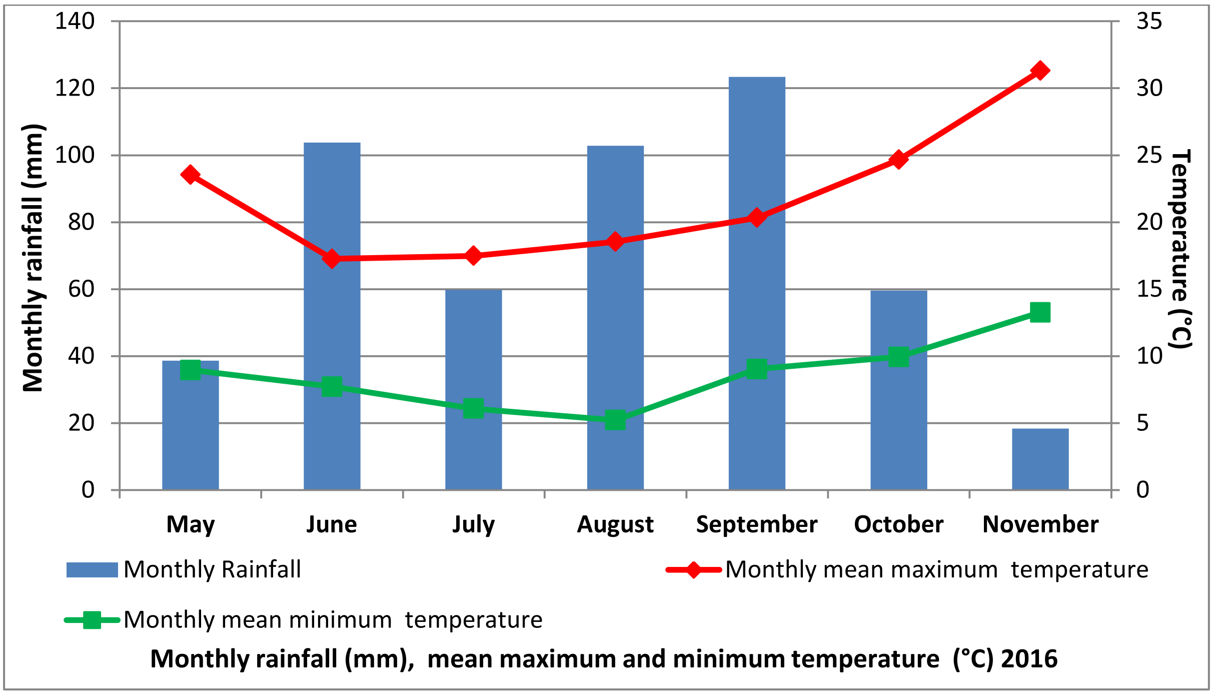

Supplementary Figure 3. Monthly rainfall (mm) and mean maximum and minimum temperatures at Narrabri during 2016 growing season.

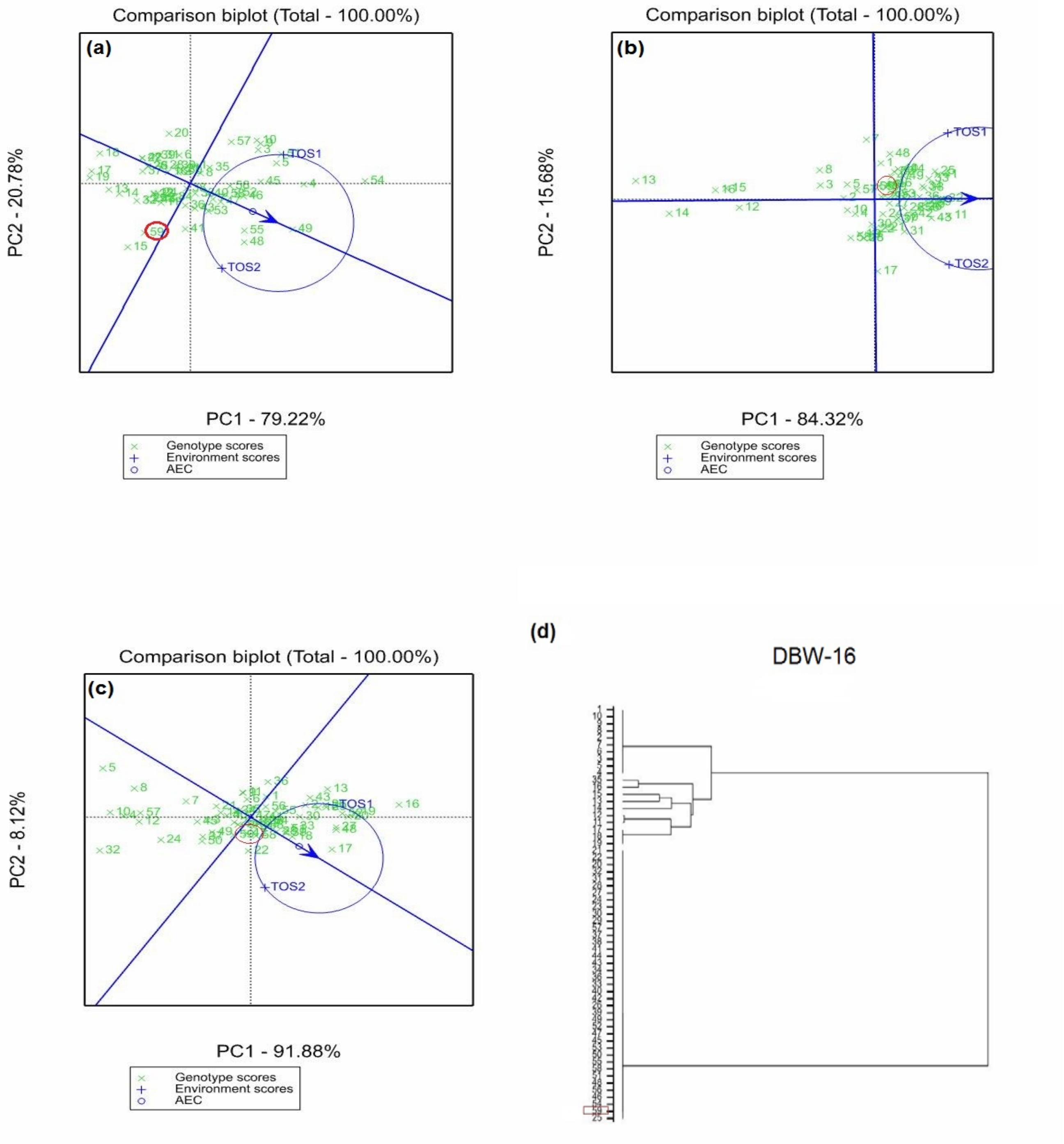

Supplementary Figure 4. Comparison of recurrent parent DBW-16 (encircled red) and progenies based on mean performance and stability across the two environments (E1, optimal sowing; E2, delayed sowing - heat stressed) for (a) screening percentage, (b) thousand kernel weight (TKW), and (c) grain yield. A dendrogram constructed using DNA diversity is given in part (d).

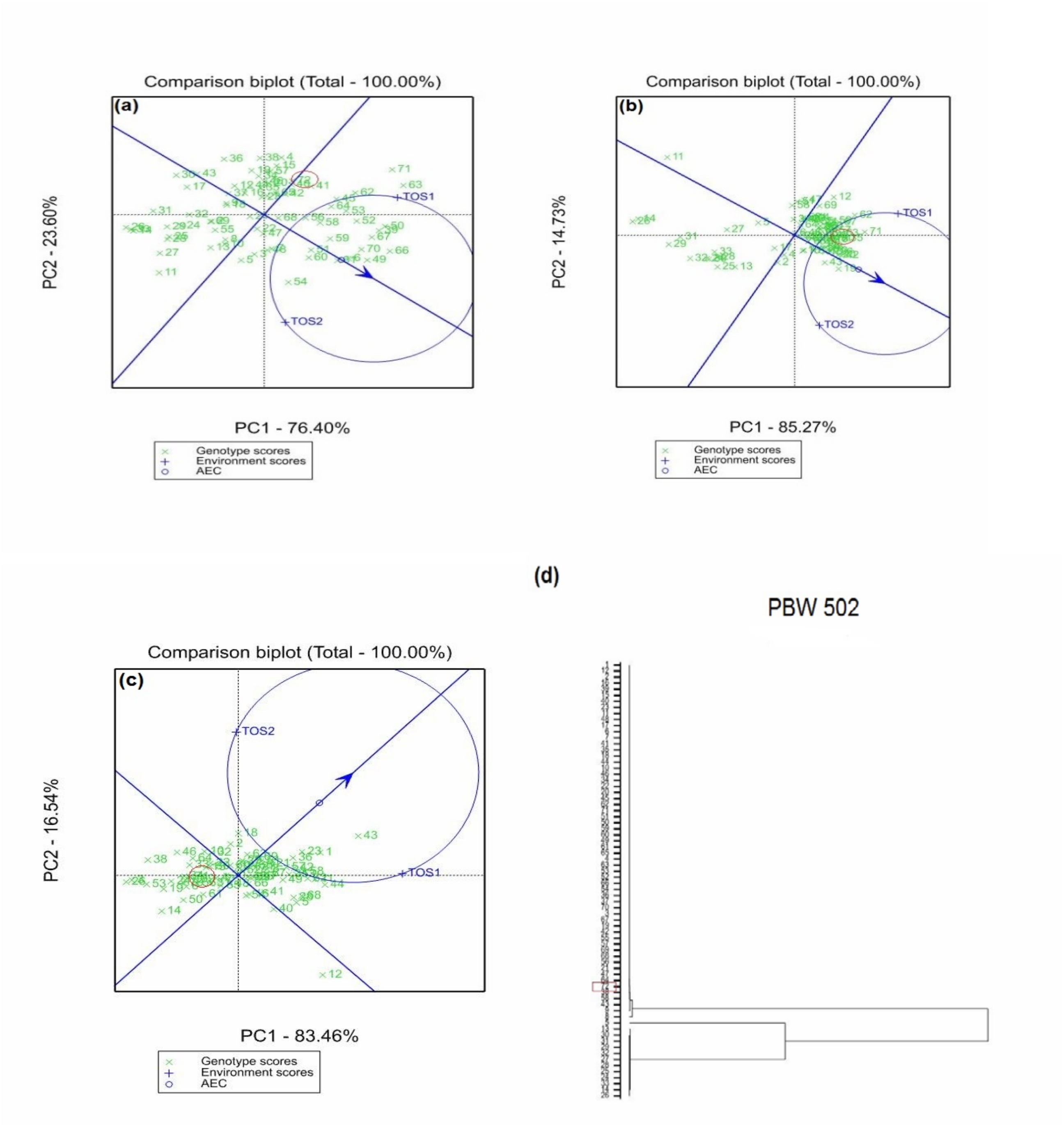

Supplementary Figure 5. Comparison of recurrent parent PBW 502 (encircled red) and progenies based on mean performance and stability across the two environments (E1, optimal sowing; E2, delayed sowing - heat stressed) for (a) screening percentage, (b) thousand kernel weight (TKW), and (c) grain yield. A dendrogram constructed using DNA diversity is given in part (d).

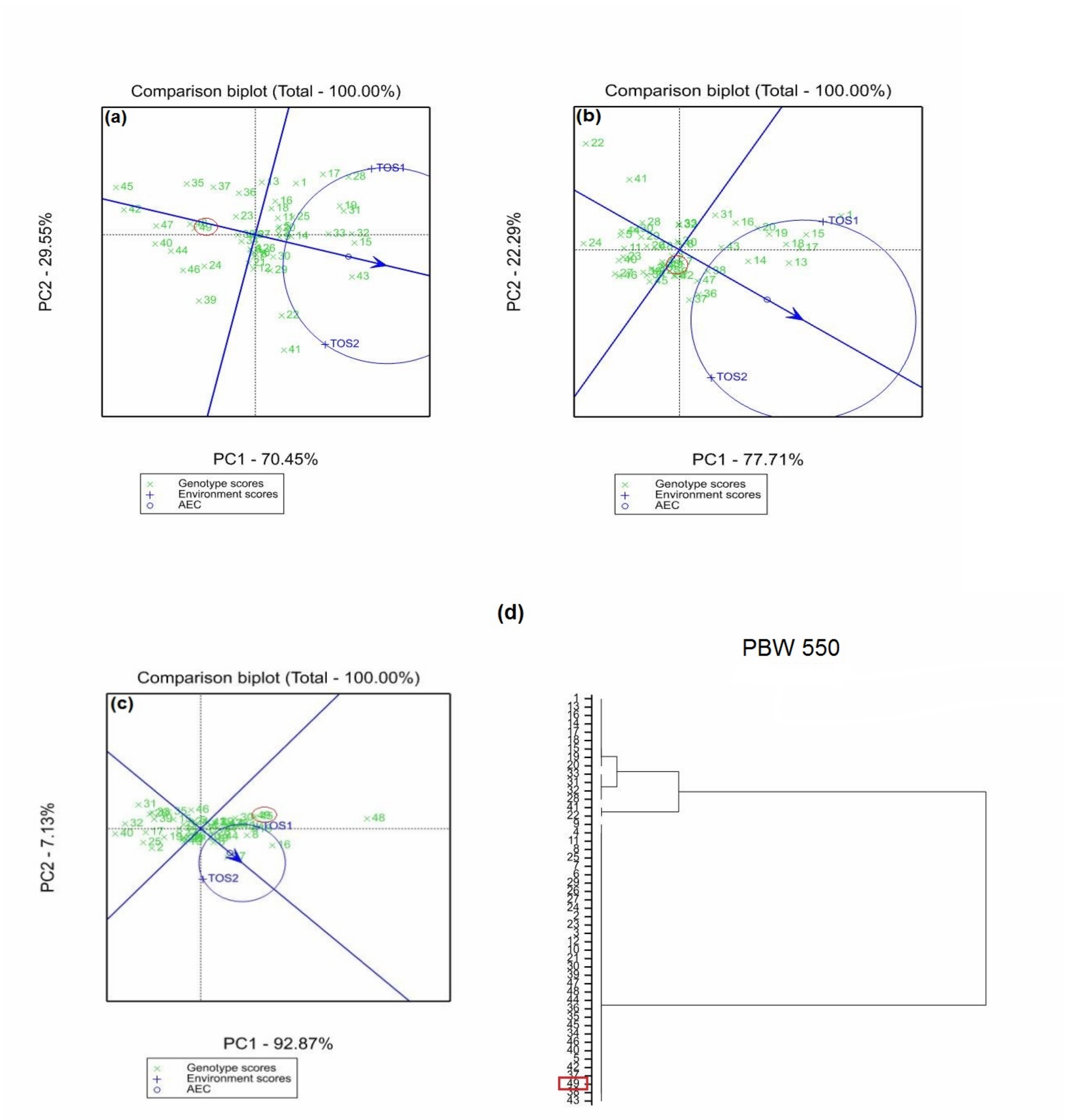

Supplementary Figure 6. Comparison of recurrent parent PBW 550 (encircled red) and progenies based on mean performance and stability across the two environments (E1, optimal sowing; E2, delayed sowing - heat stressed) for (a) screening percentage, (b) thousand kernel weight (TKW), and (c) grain yield. A dendrogram constructed using DNA diversity is given in part (d).

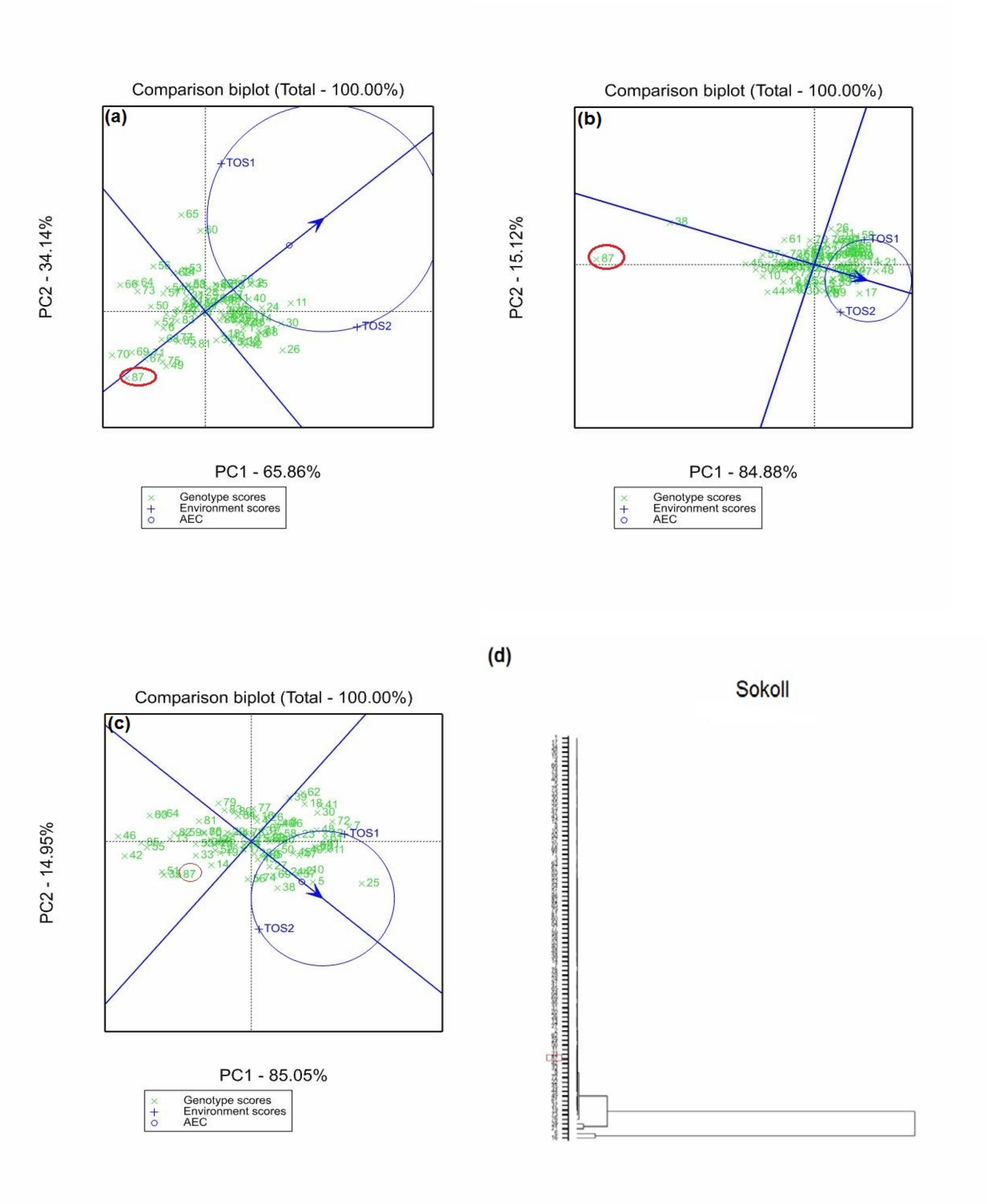

Supplementary Figure 7. Comparison of recurrent parent Sokoll (encircled red) and progenies based on mean performance and stability across the two environments (E1, optimal sowing; E2, delayed sowing - heat stressed) for (a) screening percentage, (b) thousand kernel weight (TKW), and (c) grain yield. A dendrogram constructed using DNA diversity is given in part (d).

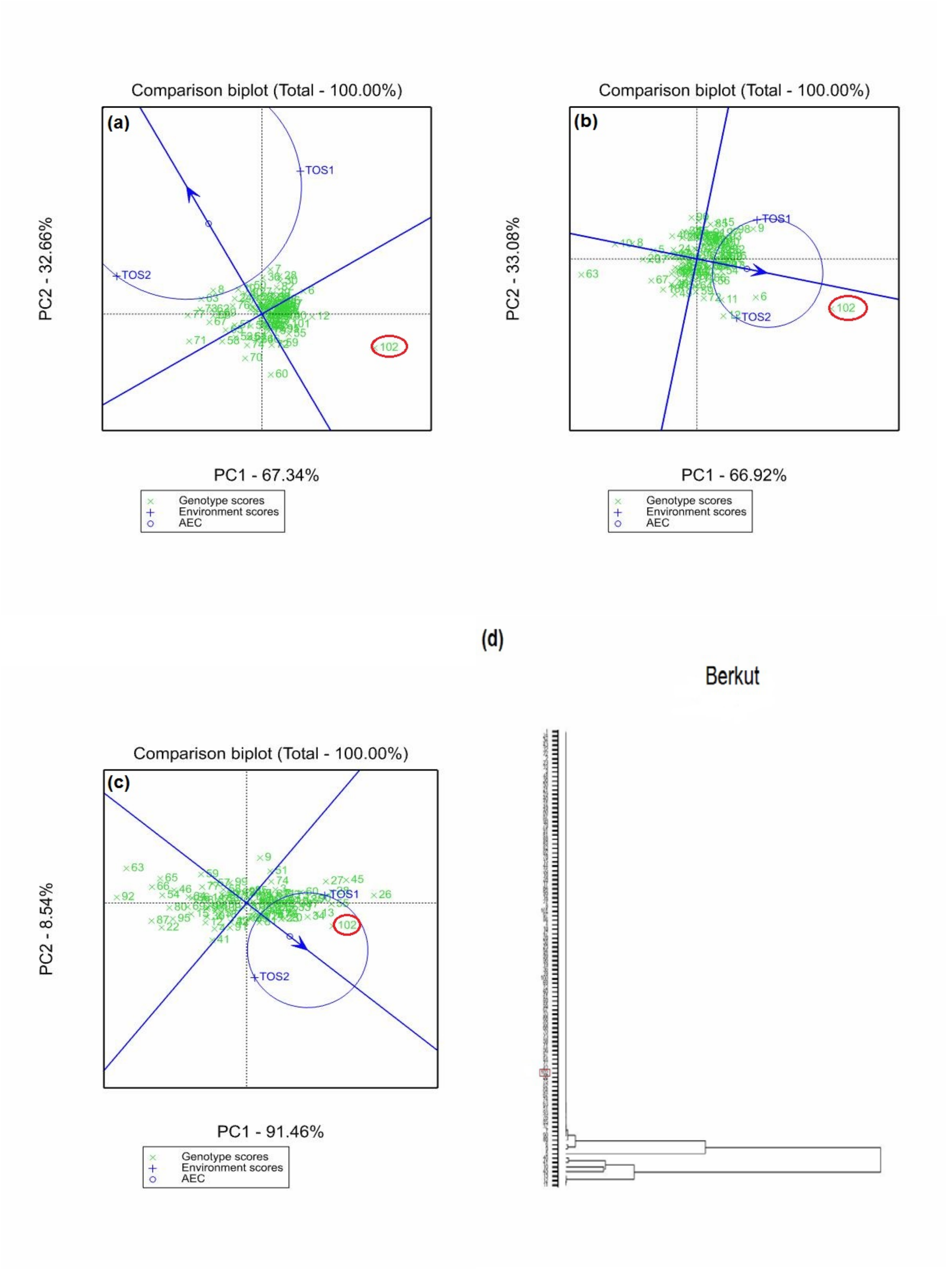

Supplementary Figure 8. Comparison of recurrent parent Berkut (encircled red) and progenies based on mean performance and stability across the two environments (E1, optimal sowing; E2, delayed sowing - heat stressed) for (a) screening percentage, (b) thousand kernel weight (TKW), and (c) grain yield. A dendrogram constructed using DNA diversity is given in part (d).
